# Nationwide and long-term molecular epidemiologic studies of mumps viruses that circulated in Japan between 1986 and 2017

**DOI:** 10.3389/fmicb.2022.728831

**Published:** 2022-10-28

**Authors:** Minoru Kidokoro, Teiichiro Shiino, Tomohiro Yamaguchi, Eri Nariai, Hiroe Kodama, Keiko Nakata, Takako Sano, Keiko Gotou, Tomoko Kisu, Tomomi Maruyama, Yumani Kuba, Wakako Sakata, Teruaki Higashi, Naoko Kiyota, Takashi Sakai, Shunsuke Yahiro, Akira Nagita, Kaori Watanabe, Chika Hirokawa, Hirotsune Hamabata, Yoshiki Fujii, Miwako Yamamoto, Hajime Yokoi, Misako Sakamoto, Hiroyuki Saito, Chihiro Shibata, Machi Inada, Misako Fujitani, Hiroko Minagawa, Miyabi Ito, Akari Shima, Keiko Murano, Hiroshi Katoh, Fumihiro Kato, Makoto Takeda, Shigeru Suga, The Surveillance Team for Mumps Virus in Japan

**Affiliations:** ^1^Department of Quality Assurance, Radiation Safety, and Information Management, National Institute of Infectious Diseases, Tokyo, Japan; ^2^Center for Clinical Sciences, National Center for Global Health and Medicine, Tokyo, Japan; ^3^Public Hygiene Division, Gifu Prefectural Tono Region Public Health Center, Tajimi, Japan; ^4^Department of Health and Food Safety, Ishikawa Prefectural Institute of Public Health and Environmental Science, Kanazawa, Japan; ^5^Division of Virology, Osaka Institute of Public Health, Osaka, Japan; ^6^Division of Microbiology, Kanagawa Prefectural Institute of Public Health, Chigasaki, Japan; ^7^Division of Virology, Ibaraki Prefectural Institute of Public Health, Mito, Ibaraki, Japan; ^8^Virus Research Center, Clinical Research Division, Sendai National Hospital, Sendai, Japan; ^9^Department of Infectious Diseases, Gifu Prefectural Research Institute for Health and Environmental Sciences, Kakamigahara, Japan; ^10^Department of Medical Microbiology and zoology, Okinawa Prefectural Institute of Health and Environment, Uruma, Japan; ^11^Kitakyushu City Institute of Health and Environmental Sciences, Kitakyushu, Japan; ^12^Department of Microbiology, Kumamoto Prefectural Institute of Public-Health and Environmental Science, Uto, Japan; ^13^Department of Pediatrics, Mizushima Central Hospital, Kurashiki, Japan; ^14^Virology Section, Niigata Prefectural Institute of Public Health and Environmental Sciences, Niigata, Japan; ^15^Awase Daiichi Clinic, Okinawa, Japan; ^16^Division of Biological Science, Hiroshima City Institute of Public Health, Hiroshima, Japan; ^17^Health Science Division, Chiba City Institute of Health and Environment, Chiba, Japan; ^18^Department of Microbiology, Akita Prefectural Research Center for Public Health and Environment, Akita, Japan; ^19^Virology and Epidemiology Division, Nara Prefecture Institute of Health, Sakurai, Japan; ^20^Laboratory of Virology, Aichi Prefectural Institute of Public Health, Nagoya, Japan; ^21^Microbiology Division, Saga Prefectural Institute of Public Health and Pharmaceutical Research, Saga, Japan; ^22^Department of Virology III, National Institute of Infectious Diseases, Tokyo, Japan; ^23^Department of Pediatrics, National Hospital Organization Mie National Hospital, Tsu, Japan

**Keywords:** mumps virus, molecular epidemiology, whole-genome sequencing, next-generation sequencing, phylogenetic analyses, genotype, Japan

## Abstract

In Japan, major mumps outbreaks still occur every 4–5 years because of low mumps vaccine coverage (30–40%) owing to the voluntary immunization program. Herein, to prepare for a regular immunization program, we aimed to reveal the nationwide and long-term molecular epidemiological trends of the mumps virus (MuV) in Japan. Additionally, we performed whole-genome sequencing (WGS) using next-generation sequencing to assess results from conventional genotyping using MuV sequences of the small-hydrophobic (SH) gene. We analyzed 1,064 SH gene sequences from mumps clinical samples and MuV isolates collected from 25 prefectures from 1986 to 2017. The results showed that six genotypes, namely B (110), F (1), G (900), H (3), J (41), and L (9) were identified, and the dominant genotypes changed every decade in Japan since the 1980s. Genotype G has been exclusively circulating since the early 2000s. Seven clades were identified for genotype G using SH sequence-based classification. To verify the results, we performed WGS on 77 representative isolates of genotype G using NGS and phylogenetically analyzed them. Five clades were identified with high bootstrap values and designated as Japanese clade (JPC)-1, -2, -3, -4, -5. JPC-1 and -3 accounted for over 80% of the total genotype G isolates (68.3 and 13.8%, respectively). Of these, JPC-2 and -5, were newly identified clades in Japan through this study. This is the first report describing the nationwide and long-term molecular epidemiology of MuV in Japan. The results provide information about Japanese domestic genotypes, which is essential for evaluating the mumps elimination progress in Japan after the forthcoming introduction of the mumps vaccine into Japan’s regular immunization program. Furthermore, the study shows that WGS analysis using NGS is more accurate than results obtained from conventional SH sequence-based classification and is a powerful tool for accurate molecular epidemiology studies.

## Introduction

Mumps is a common and highly infectious disease caused by the mumps virus (MuV), and patients infected with this virus mainly present with fever and painful swelling of the salivary glands. It may cause serious complications, such as aseptic meningitis, orchitis, pancreatitis, encephalitis or deafness.

Mumps is a vaccine-preventable disease. Mumps-containing vaccines (MCVs) were introduced in 123 countries (WHO, 2020). In several of these countries, two-dose measles-mumps-rubella (MMR) vaccines were adopted into their national immunization programs. Particularly, in most developed countries whose immunization coverages were maintained at high ratios, mumps incidence significantly declined after the introduction of MCVs into the routine immunization program (Galazka et al., 1999). However, these countries suffered from recurrent mumps outbreaks in the 2000s (Watson-Creed et al., 2006; Centers for Disease and Prevention, 2006a,b; Kutty et al., 2014). One of the main causes of these resurgences were most likely due to waning immunity of mumps vaccines (Cohen et al., 2007; Rubin et al., 2008). In addition, recent studies suggested the possibility of antigenic mismatch between currently circulating wild strains and vaccine strains (Rubin et al., 2006; Santak et al., 2015; Gouma et al., 2018; May et al., 2018).

**Figure 1:**
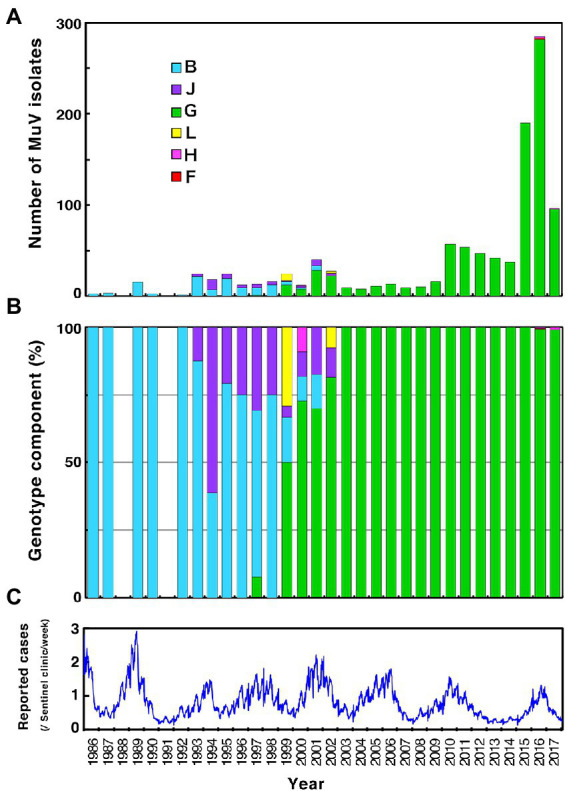
Chronological distribution of mumps virus (MuV) genotypes identified in this study and the epidemic curve of mumps in Japan from 1989 to 2017. Identified genotypes are differentiated by color. **(A)** The number of MuV isolates according to the year of isolation. **(B)** The component ratio of identified genotypes in each year. **(C)** The reported mumps cases from sentinel pediatric clinics nationwide each week.

In Japan, domestic monovalent mumps vaccines were approved from 1980 to 1991, Urabe Am9 and Hoshino strains in 1980, Torii strain in 1982, Miyahara strain in 1989, and NK-M46 strain in 1991, respectively. Coupled with that, the mumps vaccines were introduced as voluntary immunization in 1981. In 1989, a domestically produced MMR vaccine was introduced into the national routine immunization program. However, as post-vaccine aseptic meningitis caused by the Urabe Am9 strain contained in the MMR vaccine was observed at a high frequency (16.6 cases per 10,000 recipients; Kimura et al., 1996), the MMR vaccine was withdrawn in 1993 (Nagai et al., 2007; Baba et al., 2011). Thereafter, two monovalent mumps vaccines, Hoshino and Torii, have been used on a voluntary basis till date. Although two doses of mumps vaccines given at age 1 year and preschool (5–6 years old) are recommended in Japan, the mumps vaccination coverage remains low (30–40%; Baba et al., 2011; NIID, 2016), since the vaccination fee is not free for a vaccinee. Therefore, large-scale mumps outbreaks continue to occur every 4–5 years in Japan (Figure 1C; NIID, 2013; Kuba et al., 2017). Meanwhile, discussion on the introduction of mumps vaccine in routine immunization program is in progress in Japan. Responding to this situation, information about Japanese domestic genotypes to date is necessary for discerning the circulating MuVs whether an indigenous or an imported strains in order to evaluate the mumps elimination progress in Japan after introduction of the mumps vaccine into Japan’s regular immunization program.

MuV, a member of the genus *Rubulavirus* of the family Paramyxoviridae, has a non-segmented, negative-sense, single-stranded RNA genome of 15,384 nucleotides (nts). The MuV genome has an extracistronic leader sequence of 55 nts at the 3′ end and a trailer sequence of 24 nts at the 5′ end. The genome contains seven tandemly arrayed genes, namely the nucleocapsid (N), P/V/I, matrix (M), fusion (F), small-hydrophobic (SH), hemagglutinin-neuraminidase (HN), and large (L), which encode nine viral proteins, N, P, V, I, M, F, SH, HN, and L. The SH gene is one of the most divergent regions within the MuV genome (Takeuchi et al., 1991; Amexis et al., 2003). Therefore, the 316 nts long SH gene sequences, which contain both non-coding regions and 174 nts SH protein-coding region, have been widely used for the molecular epidemiology and genotyping of MuVs (Afzal et al., 1997; Jin et al., 2005; Palacios et al., 2005).

MuVs are classified into 12 genotypes based on SH gene sequences, which are named A to N (excluding E and M; WHO, 2012). Meanwhile, since MuV is serologically monotypic, current vaccine strains, which were originated from old genotypes, for instance, Jeryl Lynn is A, Japanese vaccine strains are B, and Leningrad-3 is N, are suboptimal but still effective against phylogenetically distinct genotype G (Deeks et al., 2011; Rubin et al., 2012; Ihara, 2014; Wohl et al., 2020). The study of mumps morbidities in 4- and 5-year-old nurseries receiving one dose of Japanese monovalent mumps vaccine or MMR vaccine in during a major mumps outbreak in 1994 reported that the effectiveness of MCV ranged from 79.8 to 88.8% (Matsuoka et al., 1995). The another study of vaccine effectiveness in elementary school children receiving one dose of Japanese monovalent vaccines, Hoshino or Torii, during a mumps outbreaks in Mie prefecture reported that the effectiveness ranged from 79.1 to 90.0% (Ihara, 2014).

Earlier molecular epidemiology studies of Japanese MuVs showed that genotype B was the dominant indigenous genotype until the 1980s, and genotypes B and J subsequently co-circulated in the 1990s. Genotype G emerged at the end of the 1990s, and since 2000, it has been circulating predominantly in Japan (Inou et al., 2004; Akiyoshi and Suga, 2014; Aoki et al., 2016), which contains at least 2 subgenotypes (Inou et al., 2004; Kidokoro, 2013; Aoki et al., 2016; Kuba et al., 2017). However, in the previous studies have limitations in the research areas owing to research at the municipal level and length of the data collection period.

We performed whole-genome sequencing (WGS) in this study to precisely analyze the phylogenetic relationships of MuV isolates. Pathogen WGS by using next-generation sequencing (NGS) technology has been a key tool for chasing the spread of infectious diseases, resolving transmission patterns (Faria et al., 2018), identifying outbreak origins (Agoti et al., 2017), detecting presence of resistance mutations (Ariey et al., 2014; Toledo-Rueda et al., 2018), reassortment (Maljkovic Berry et al., 2016), pathogenicity, and novel pathogens (Chen et al., 2011; Grard et al., 2012). In mumps outbreaks, WGS analysis have provided information about throughout genomic changes possibly affecting vaccine effectiveness, diagnostic sensitivities, and greater resolution and accuracy in mapping transmission pathways (Dilcher et al., 2018; Alkam et al., 2019; Stapleton et al., 2019; Wohl et al., 2020).

This study aimed to describe nationwide and long-term (1986–2017) molecular epidemiologic trends of MuVs circulating in Japan and to obtain essential information about Japanese domestic MuV genotypes required for evaluating the mumps elimination progress in Japan after introducing the mumps vaccine into Japan’s regular immunization program.

## Materials and methods

### Clinical samples

We collected 1,064 MuV sequences obtained from 644 clinical samples and 420 MuV isolates. These samples were obtained from 19 local governmental health institutes nationwide and five local base hospitals in 24 prefectures during 1986 to 2017. The clinical samples (including 362 buccal swabs, 433 throat swabs, 119 spinal fluids) were collected from patients with clinically suspected mumps in the hospitals by doctors and sent to the local government laboratories or the National Institute of Infectious Diseases (NIID) and stored at −70°C or lower in each laboratory until they have served for analyses. The case definition for mumps includes clinical symptoms of rapid swelling of uni- or bilateral salivary glands with pain. A confirmed case was defined by the clinical symptoms plus a laboratory confirmation by detecting MuV RNA by RT-PCR from clinical samples. Among the 1,064 specimens collected, 623 were sequenced in the local government laboratories. The remaining 441 samples that were collected in the hospitals were sequenced at NIID. The phylogenetic analyses were conducted at NIID and National Center for Global Health and Medicine.

The MuV isolation and propagation were performed as previously reported (Kidokoro et al., 2011). Briefly, the supernatants of centrifuged clinical samples which were soaked in Eagle’s Minimum Essential Medium (MEM), which were supplemented with 3% fetal bovine serum, 200 μg/m streptomycin, and 200 U/ml penicillin, were inoculated onto Vero cells. The cells were cultured at 37°C and the supernatants were harvested when those cytopathic effects (CPE) became prominent. Two blind passages were performed on all CPE negative tissue cultures.

### RNA preparation, RT-PCR, and Sanger sequencing

Viral RNA was extracted from clinical samples or the culture fluids of mumps isolates by using the QIAamp Viral RNA Mini Kit (Qiagen, Tokyo, Japan) following the manufacturer’s instructions. The SH gene (the genome positions including from 6,076 to 6,803) was amplified with a set of primers SH-1 (5′-GCRACYAAAGARATCAGRAGRATC-3′) and SH-2 (5′-AGCCTTGATCATTGATCATCC-3′), using PrimeScript™ One Step RT-PCR Kit Ver.2 (TaKaRa, Kyoto, Japan) according to the manufacturer’s instructions. When the RT-PCR products were not detected, we performed a second PCR using nested primer set designed to amplify the genome positions from 6,130 to 6,689 with a set of SH-3 (5′-TCAAGYAGTGTCGAYGATCTC-3′) and SH-4 N (5′-AGCCTTGATCATTGATCATCC-3′) primers. The PCR products were sequenced using the BigDye Terminator v3.1 Cycle Sequencing Kit and 3130*xl* Genetic Analyzer (Applied Biosystems Japan, Tokyo, Japan).

### NGS

We applied the viral RNAs extracted from culture fluids of 82 MuV isolates of the current study (of these, 77 were genotype G, 5 were genotype L) to the NEBNext Ultra RNA Library Prep Kit for Illumina, followed by the NEBNext rRNA Depletion Kit (Human/Mouse/Rat; NEW ENGLAND BioLabs Japan, Inc. Tokyo, Japan) according to the manufacturer’s instructions. Thereafter, sequences were generated using paired-end sequencing on the MiSeq platform (Illumina K.K. Tokyo, Japan). The 77 strains of genotype G isolates were constituted by 21 strains which represented the subclades based on SH sequence, and the phylogenetically related 56 isolates. Of those, 31 isolates share the identical SH gene sequence. Five of the 6 genotype L strains also share the identical SH gene sequence. We used CLC Genomics Workbench version 8.0.1(CLC Bio, Qiagen, Germany) for assembly, reference mapping, and generation of consensus sequences of mumps isolates. The sequences were deposited in the DNA Data Bank of Japan (DDBJ) and the accession numbers are provided in Supplementary Table S1.

### Phylogenetic and evolutionary analyses

A total of 316 nts of the sequences containing the entire SH gene (the genome positions from 6,218 to 6,533) were used for genotyping. To verify the results of SH gene-based analysis for genotype G, we performed phylogenetic analyses by using whole-genome sequencing (WGS) through whole-genome sequence datasets (*n* = 101), excluding both terminal sequences (WGS-dt, 15,292 nts between genomic positions 56 and 15,347). In order to compare our genotype G strains with as many SH gene and WGS data as possible, we searched for the National Center for Biotechnology Information (NCBI) Genbank and retrieved 7,824 and 177 sequences of SH gene and WSG, respectively, and selected nonredundant sequences (884 strains for SH gene and 30 strains for WGS-dt) based on their sequences, isolated areas and isolated years. Regarding genotype L, we adopted the dataset of HN gene sequences for the analyses instead of the WGS sequences, because WGS data of genotype L were not available in NCBI except only one strain. Then we aligned them with the sequence data of reference strains proposed by the World Health Organization (WHO; WHO, 2012) using MUSCLE (Edgar, 2004). Phylogenetic trees of SH gene, WGS-dt and HN gene sequences were inferred by the maximum-likelihood (ML) method using the Tamura-Nei model with the gamma-distributed site (TN93 + G) for SH gene and general time reversible model with gamma-distributed site (GTR + G) for WGS-dt and Tamura 3-parameter model (T92) for HN gene as the nucleotide substitution model. The topology of the phylogenetic tree was validated by Felsenstein’s bootstrap test with 1,000 resamplings, and > =75% of the probability was considered as a significant monophyletic group or clade. The multiple alignment, ML tree inference and the substitution model selection were conducted using Molecular Evolutionary Genetics Analysis (MEGA) software version 10.1.1 (Kumar et al., 2016). To confirm the evolutionary process of MuV transmissions in Japan along with time, WGS-dt of genotype G and HN gene of genotype L were also analyzed by Bayesian Markov Chain Monte Carlo (MCMC) coalescence method using BEAST 1.9 or BEAST 2.4.7. We used the GTR + G substitution model for the WGS-dt dataset and the HKY substitution model for the HN gene dataset. Models for the clock and population were evaluated in the actual data by log marginal likelihood estimation using path sampling and stepping-stone sampling (Baele et al., 2012), and adopted a combination of relaxed clock gamma distribution model and Bayesian Skyline Plot (BSP) for the WGS-dt, and a combination of relaxed clock log normal model and Coalescent Exponential Population for the HN gene. To ensure convergence at a minimum effective sample size (ESS) of >200, MCMC chain set in 300 million and were sampled at every 30,000 generations, and removing 300 million steps as burn-in. The MCMC output was analyzed using Tracer v.1.6.0 software^1^ and the evolutionary parameters, mean times of the most recent common ancestor (tMRCAs) of the clades identified by ML tree, and the BSP-based population size changes in time were estimated for a most major clade of genotype G, JPC-1. Bayesian maximum clade credibility (BMCC) chronological tree inference was constructed using TreeAnnotator v.2.4.7 software and visualized in DensiTree v.2.0 and FigTree v.1.4.4. The list of the representative MuV isolates in this study and their accession numbers are provided in Supplementary Tables S1, S2.

### Multiple alignment of amino acid sequences of MuV viral components

Multiple alignments of the deduced amino acid sequences for the mumps virus proteins, N, V, P, M, F, SH, HN and L, of the 77 isolates of Japanese genotype G (JPC-1, 64 strains; JPC-2, 2 strains; JPC-3, 8 strains; JPC-4, 1 strain; JPC-5, 2 strains) were constructed by using GENETYX-MAC software v16.0.7 (GENETYX CORPORATION, Japan). The alignments were constructed from nonredundant amino acid sequences from each JPC. Excepting SH protein, two current Japanese mumps vaccine strains, Hoshino (AB470486) and Torii (MD217222) were aligned for reference sequences. In regard to SH protein, five additional isolates in our study (MuVi/Mizushima#06.JPN/43.06[G], MuVi/Mie#B11.JPN/34.11[G], MuVs/Kumamoto#175.JPN/53.12[G], MuVi/Mie#D10.JPN/12.14[G], MuVi/Kanagawa259.JPN/27.15[G]), that harbored truncated forms of SH protein, were aligned with 77 isolates.

## Results

### Specimen descriptions

**Figure 2:**
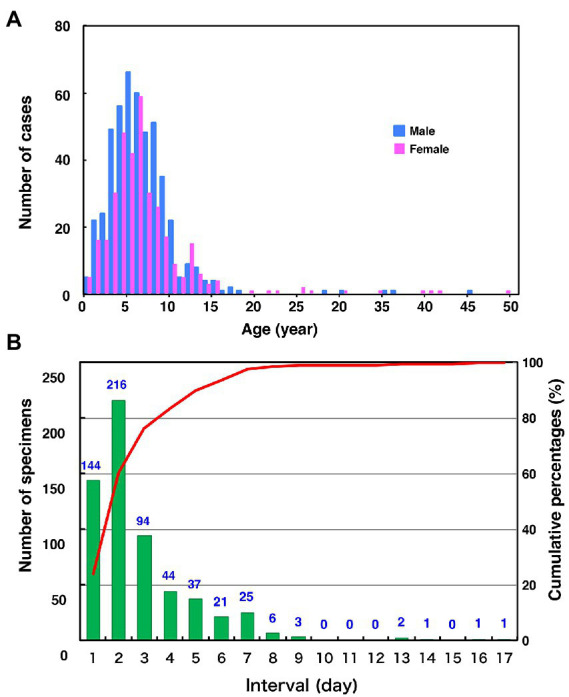
Distributions of age by gender **(A)** and the number of days between the onset of mumps and sample collection **(B)** in this study. **(A)** Blue and the red bars indicate the number of male and female mumps cases, respectively. **(B)** The green bars and the red line show clinical sample numbers and cumulative percentage by interval days between the onset of mumps and sample collection, respectively.

The age distribution of the 827 cases, whose epidemiological backgrounds are evident, range from 0 to 49 years old (median 6.0-year-old [IQR, 4.0–8.0 year-old]; Figure 2A). Most of the patients (89.8%) were children aged 10 years or younger. Unexpectedly, although the distribution also showed a minor peak during 11 and 15 years old, the proportion of the adolescents and adults aged 16 to 49 years was relatively low (2.5%). One of the reasons for this is that most specimens might have been collected from pediatric clinics. Among the 672 patients whose symptoms were clear, the most common symptoms were parotitis (499 cases, 74.3%) and aseptic meningitis (166 cases, 24.7%). There were five cases (0.7%) of encephalitis or encephalopathy. In this study, orchitis was reported in only two cases (0.3%) as the vast majority of patients were children under 10 years of age. Although the mumps vaccine histories of over 80% of the patients were not described (887 cases, 84.2%), the previously vaccinated or unvaccinated cases were 96 (9.1%) and 81 (7.7%), respectively. The male-to-female ratio of patients was 58.2% versus 41.8%. These results show similar patterns with the data on age- and gender-specific incidences of mumps in Japan, which were from the Infectious Agents Surveillance Report, whose data were collected from approximately 3,000 sentinel pediatric clinics nationwide.^2^ The time intervals between the onset of mumps and sampling of the specimens ranged from 0 to 16 days (median 1.0 d [IQR, 0.0–2.0 d]; Figure 2B). Notably, >75% MuV-positive specimens (454 [76.3%]) were sampled within 2 d of onset. These results highlight that mumps clinical samples should be collected within 2 d of mumps symptom onset, and support previous findings (Rota et al., 2013; Golwalkar et al., 2018; Shah et al., 2018).

### Genotype identification in Japan from 1986 to 2017

**Figure 3:**
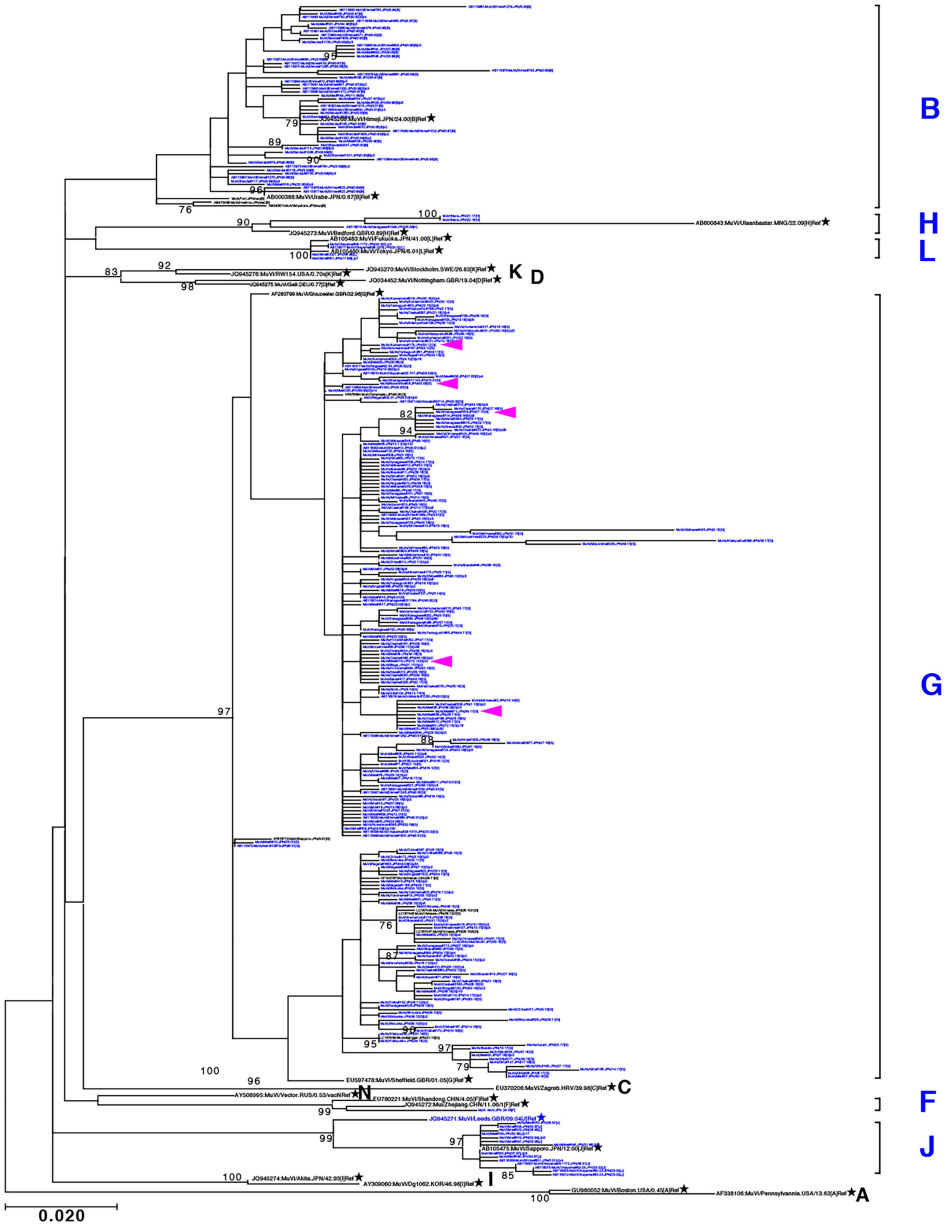
Maximum-likelihood phylogenetic tree inferred by SH gene sequence data of mumps virus (MuV) isolates identified in this study, the World Health Organization (WHO) reference strains, and related strains obtained from Data Bank of Japan (DDBJ). MuV strains obtained in this study are indicated by blue letters. WHO reference strains are indicated by black letters and marked with stars (★). Related strains are indicated by black letters. Bootstrap values >75% are indicated on the trees. Alphabets A to N indicate the genotypes of MuVs proposed by WHO; the six genotypes shown on the right side in blue letters are the genotypes identified in this study. Genotype A reference sequences were employed as the outgroup. Magenta arrow-heads denote the strains harboring the SH genes with truncated open reading frames.

**Figure 4:**
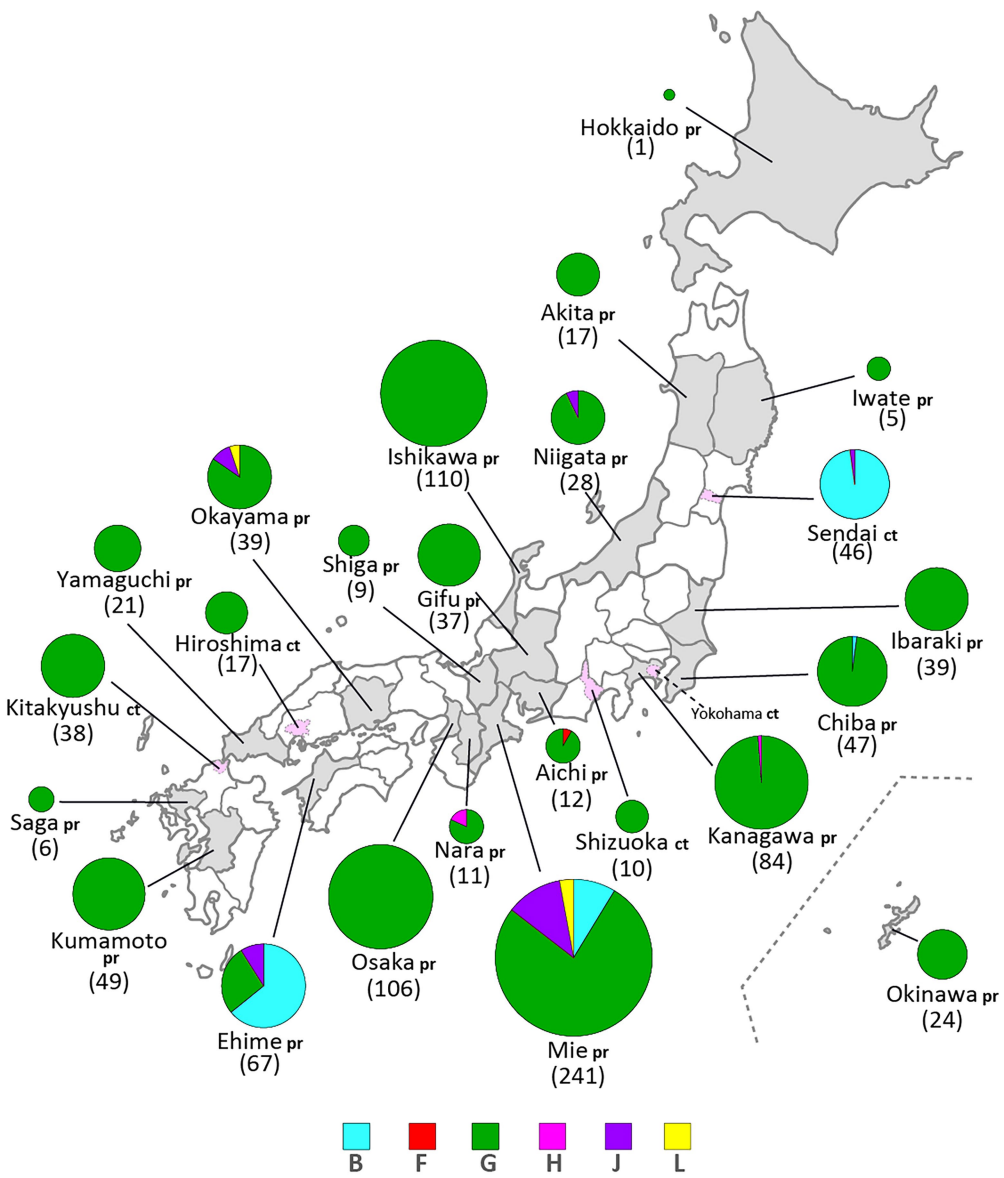
Geographical distributions of mumps virus (MuV) genotypes identified in this study from 1986 to 2017. Identified genotypes are differentiated by color. The numbers in brackets refer to the total numbers of the MuVs isolated from the prefectures (pr) or cities (ct). The administrative divisions where MuVs were isolated are color coded: grey-colored area for prefecture, pink-colored area surrounded by dashed line for city.

**Figure 5:**
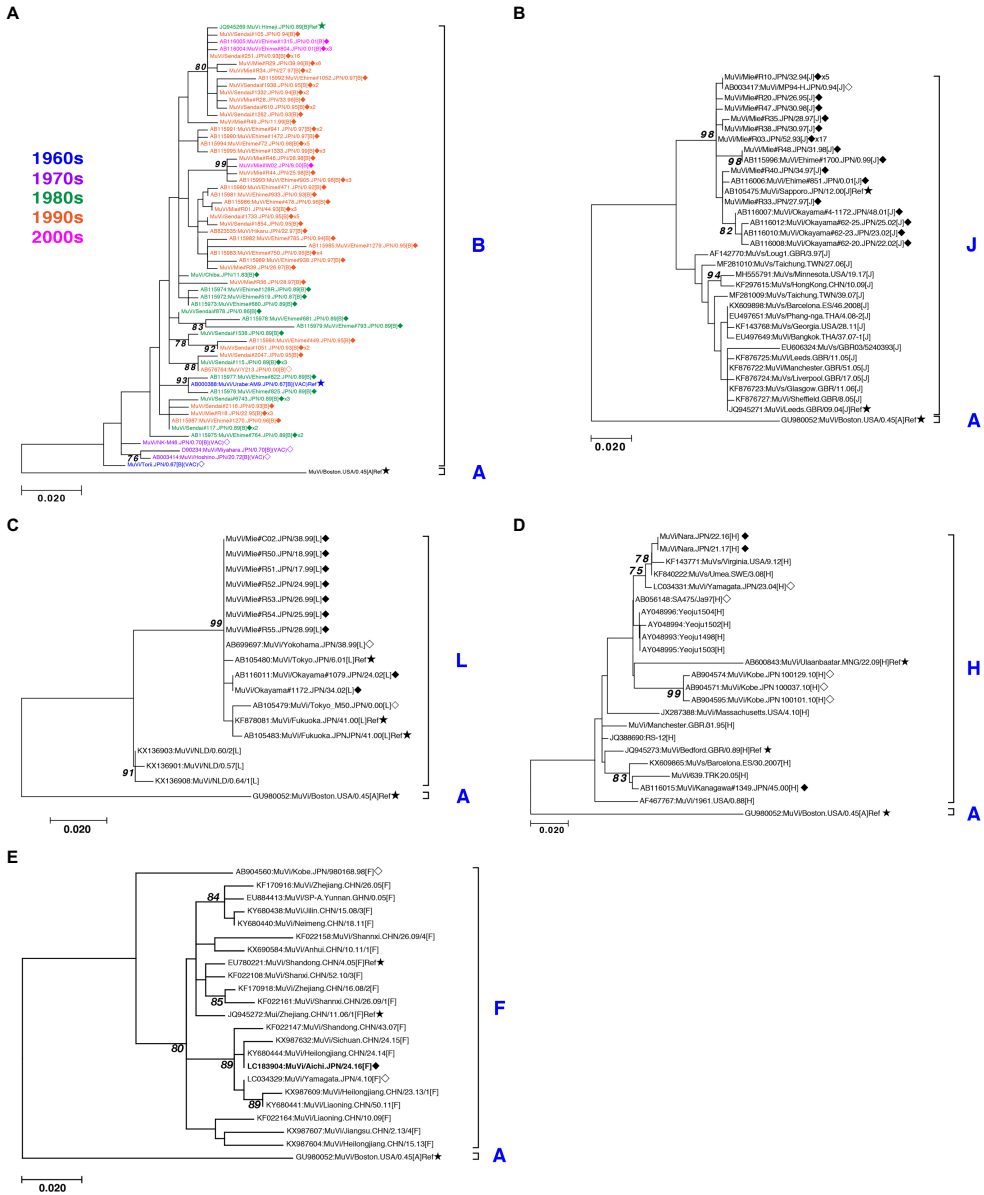
Maximum-likelihood phylogenetic tree of the genotype B **(A)**, J **(B)**, L **(C),** H **(D)**, and F **(E)** based on the SH gene sequences. The World Health Organization (WHO) reference strains are indicated by stars (★). The strains collected in this study are indicated by a filled rhombus (◆). The domestic related strains are indicated by an empty rhombus (◇). The trailing number of the strain name indicates the number of isolates with identical sequences in the study. Bootstrap values >75% are indicated on the trees. The genotype B strains collected in the study are color coded according to the decade of isolation (1960s in blue, 1970s in purple, 1980s in green, 1990s in orange, and 2000s in magenta).

Through phylogenetic analyses based on 1,064 SH gene sequences, six genotypes, namely B, F, G, H, J, L, (110 [10.3%], 1 [0.1%], 900 [84.6%], 3 [0.3%], 41 [3.9%], and 9 strains [0.8%], respectively) were identified (Figure 3). The 353 selected representative MuV strains are listed in Supplementary Table S1. Chronological changes and geographical distribution of genotypes identified in the study are shown in Figures 1, 4. In the 1980s, genotype B was in exclusive circulation in Japan (Figure 1A,B) because it is a classic Japanese indigenous genotype, as with all Japanese vaccine strains, whose parental strains were isolated in the late 1960s or early 1970s (Figure 5A). Therefore, the genotype B isolates in the 1980s and 90s were classified into distinct clades against Japanese vaccine strains, except for Urabe strains (Figure 5A). Genotype B viruses continued to circulate until 2001 (Figure 1A,B).

Genotype J first emerged in 1993 and co-circulated in Japan with genotype B until the early 2000s (Figure 1A,B) in Japan. Genotype J strains have also been reported in several other countries, including Singapore, United Kingdom, Taiwan, Malaysia, Spain, Ireland and Thailand (Jin et al., 2015). However, the Japanese strains are genetically distinct from the foreign strains, whose inter-clade divergences ranged from 3.5 to 7.6% (Figure 5B). Additionally, the Japanese strains are highly homologous to each other, with a maximum intra-clade divergence of 2.8%, compared to that of the foreign strains (4.7%).

Genotype L circulated in Japan from 1999 to 2002 and disappeared after a short period (Figure 1A,B). However, the genotype L viruses caused nationwide epidemics, because they were isolated from various parts of Japan, such as Tokyo, Mie, Okayama, Fukuoka prefectures and, Yokohama city in Kanagawa prefecture (Figure 5C), even though only two prefectures, namely Mie and Okayama, were registered in the study (Figure 4). The sequences formed a single clade with high homology, whose divergences ranged from 0.0 to 1.3%, and were evidently divergent from Netherlands strains with the high bootstrap values (Figure 5C). To confirm the result of ML phylogenetic analysis based on the SH genes, we performed time-scaled Bayesian evolutionary analyses along with ML phylogenetic analysis based on the datasets of 11 HN gene sequences (6 Japanese strain and 5 reference strains) of genotype L strains (Supplementary Figures S1A,B). The resulting trees described that genotype L strains were divided into two phylogenetically distant clades, Japanese clade and Netherlands clade with high bootstrap values (100%), which showed similar tree topologies with that of the SH based ML tree (Supplementary Figures S1A,B). We also estimated mean times to the most common recent ancestor (tMCRA) of these two clades, which were 1996.70 (95% highest posterior density (HPD): 1999.10–1993.38) for Japanese clade, and 1955.92 (95% HPD: 1957.50–1951.91) for Netherlands clade, respectively (Supplementary Table S3). The tMCRA of all genotype L strains was 1954.83 (95%HPD: 1957.49–1944.41). These data suggest that the Japanese genotype L viruses could not be directly related with European genotype L strains.

In our study, Japanese genotype H viruses were identified only three strains, one of which was isolated from Kanagawa Prefecture in 2000 (MuVi/Kanagawa.JPN/45.00). The others were isolated from Nara Prefecture from 2016 to 2017 (MuVs/Nara.JPN/22.16, MuVs/Nara.JPN/21.17; Figures 1A,B, 4, 5D). These isolates were classified into two distinct clades (Figure 5D). Two isolates from Nara Prefecture that shared an identical sequence were classified into a clade, including isolates from Asian countries, such as Korea, Mongolia, and Japan, despite the fact that the most closely related strain was the isolate from Sweden (KF840222; Figure 5D). Almost all Japanese genotype H isolates were classified in this clade. Moreover, MuVi/Kanagawa.JPN/45.00 was classified into distinct clades, which primarily included strains isolated from the Western and the Middle Eastern countries, including United Kingdom, Spain, United States, Turkey, and Iran. This is the only isolate of the clade in Japan thus far.

The singular isolate of genotype F in this study was isolated from Aichi Prefecture in 2016 (MuVi/Aichi.JPN/24.16), whose sequence was identical to that of the Chinese isolate in 2014 (KY680444, Figure 5E). The results suggest that this could be an imported case from China, even though epidemiologic links were unclear.

**Figure 6:**
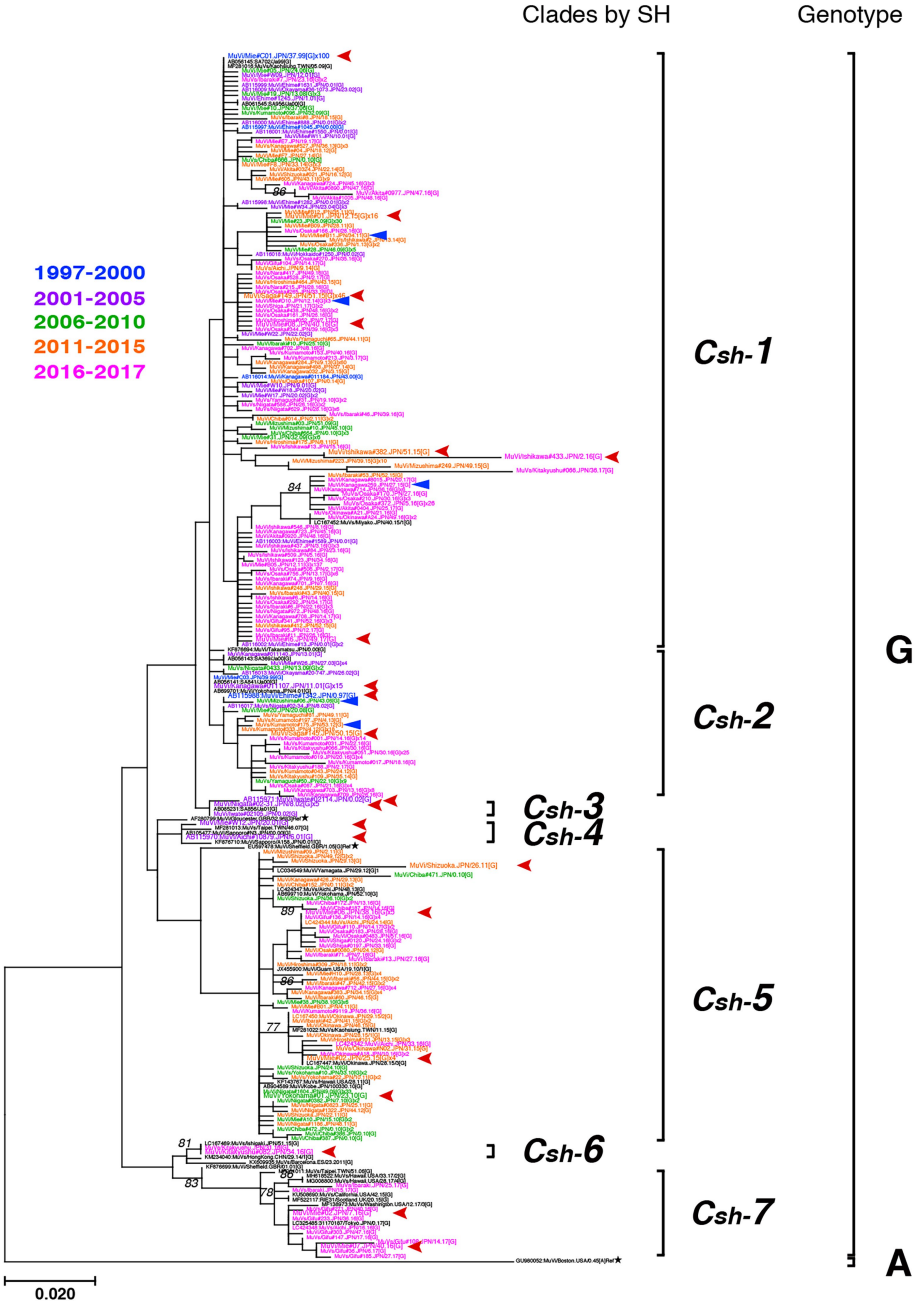
Maximum-likelihood phylogenetic tree of the genotype G strains based on the SH gene sequences. The World Health Organization (WHO) reference strains are indicated by stars (★). The related strains obtained from the DNA Data Bank of Japan (DDBJ) are indicated in black letters. The strains collected in this study are color coded according to isolated years (1997–2000 in blue, 2001–2005 in purple, 2006–2010 in green, 2011–2015 in orange, and 2016–2017 in magenta). Blue arrow-heads denote the strains harboring the truncated form SH genes. Red arrow-heads denote the strains applied to whole-genome sequencing (WGS) using next-generation sequencing (NGS), which represent each clade.

Genotype G was the most frequently isolated genotype in our study (900 isolates out of 1,064 isolates, 84.6%) as it was predominant in the last two decades in Japan, and our surveillance network began to work at full scale during this period. Although previous studies have reported that the Japanese genotype G consists of two major clades (Akiyoshi and Suga, 2014; Aoki et al., 2016; Kuba et al., 2017), we identified at least seven clades in this study using SH-based classification on the basis of the topology of the phylogenetic tree, designated Csh-1, -2, -3, -4, -5, -6, and -7 (Figure 6). However, as most bootstrap values of each clade were <75%, the topology of the phylogenetic tree could be inaccurate. In effect, topologies of phylogenetic trees varied depending on strain combinations of genotype G (Figures 3, 6).

### Detailed analyses of genotype G

**Figure 7:**
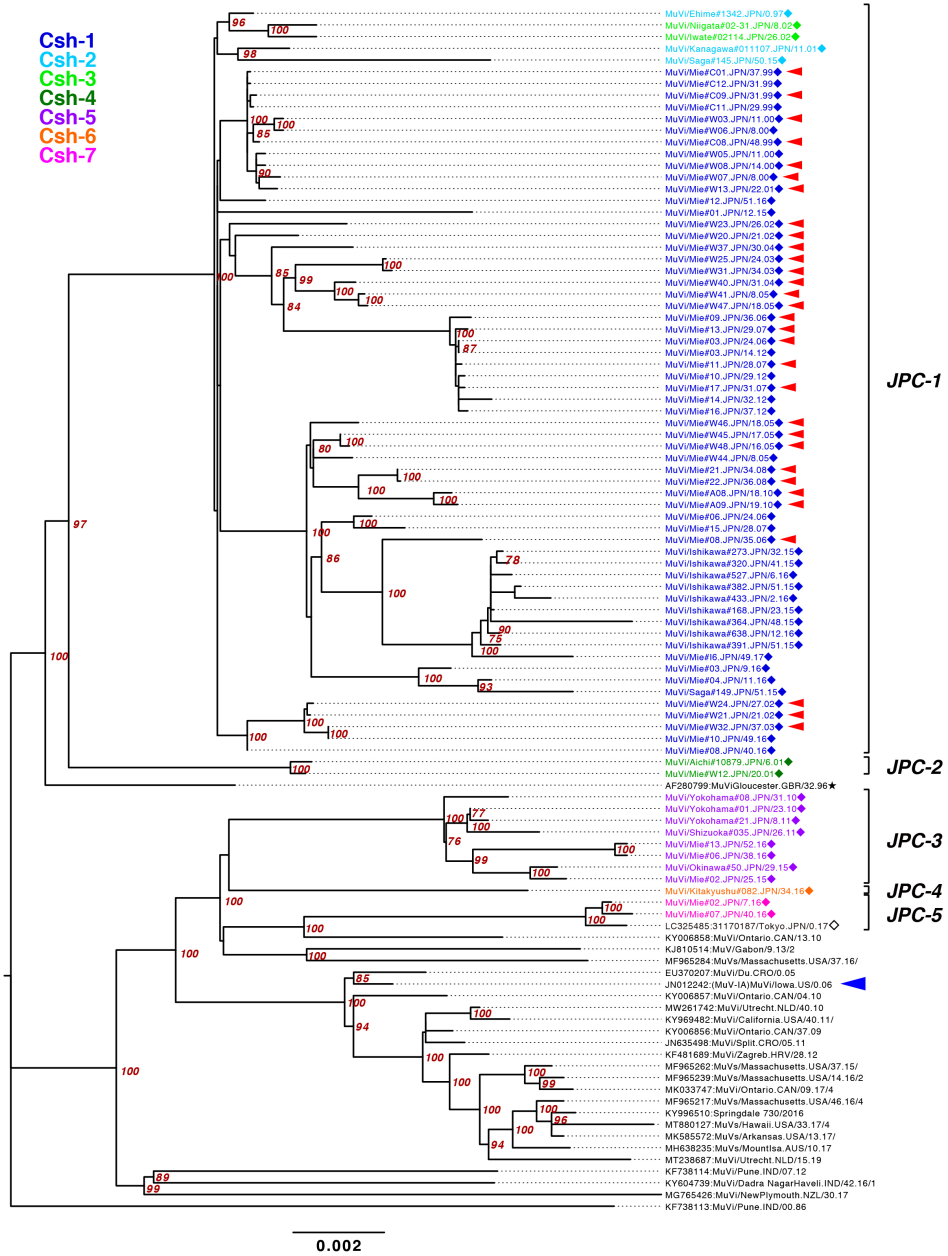
Maximum-likelihood phylogenetic tree of the genotype G strains based on WGS-dt. The WHO reference strains are indicated by stars (★). The strains collected in this study are indicated by a filled rhombus (◆). The domestic related strains are indicated by an empty rhombus (◇). Bootstrap values >75% are indicated on the trees. The isolates of this study are color coded according to the provisional clade based on the SH sequences: blue for Csh-1, cyan for Csh-2, green for Csh-3, moss green for Csh-4, purple for Csh-5, orange for Csh-6, and red for Csh-7. Red arrow-heads denote the strains sharing the absolute identical SH gene sequence. Red arrow-heads denote the strains harboring an absolutely identical SH gene sequence. Blue arrow-head denotes the isolate of the large outbreak in Iowa, USA in 2006.

To establish the phylogenetic relationships among the seven clades based on SH sequences, we performed WGS using NGS on 77 strains of genotype G isolates, which contained 21 strains representing each clade by SH (Figure 6) and 56 related strains isolated in the study. They were analyzed by using both ML phylogenetic method and Bayesian MCMC method based on WGS data excluding leader and trailer sequences (WGS-dt, 15,292 nts between genomic positions 56 and 15,292; Figure 7; Supplementary Figure S2; Supplementary Table S2). Through the MCMC analyses, we also estimated the tMCRA of each clades of Japanese genotype G (Supplementary Table S3).

The bootstrap values in ML tree of the WGS-dt were much higher (> = 97%) than those obtained in SH gene detaset. The posterior probabilities of the Bayesian tree were also sufficiently high (> = 0.99). The both resulting trees inferred from the WGS-dt dataset exhibited similar topologies, which showed that Japanese genotype G strains were divided into two major clades and each clade was further divided into two and three minor clades, respectively. Consequently, the seven clades based on SH sequences were consolidated into five clades, which were designated Japanese clade (JPC)-1, -2, -3, -4, and -5, numbered in order of emerging times of the first isolate of each clade for convenience. As a result, the three SH-based clades, Csh-1 to -3, were classified as JPC-1 (Figure 7; Supplementary Figure S2).

In order to clarify the phylogenetic relationships between the JPCs and globally distributed genotype G strains, we constructed phylogenetic tree from the dataset of SH sequences from 867 genotype G sequences retrieved from NCBI and 30 representative strains this study, 28 of which were from WGS-dt data (Supplementary Figure S3).

The JPC-1, tentatively called “Gw,” was the first identified and the most abundant clade in Japan (726 isolates, 80.7% of genotype G). The first identified JPC-1 strain was isolated from Ehime Prefecture in 1997 (MuVi/Ehime#1342.JPN/0.97). The clade subsequently became the great majority of the MuV isolates during the next 5 years. The tMCRA of JPC-1 was 1992.84 (95% HPD: 1996.26–1988.61; Supplementary Figure S2). Some strains, which isolated in foreign regions, Mainland China, Taiwan, North America, were also classified into the JPC-1, all of which were isolated after the tMCRA of JPC-1 (Supplementary Figure S3; Supplementary Table S3). Of these, three Taiwan isolates (MF281016, MF281017, MF281023) were isolated from a Japanese, a Thailander, and a Korean, respectively (Cheng and Liu, 2018). Two isolates of United States (FJ959106, FJ959107) were isolated from outbreaks occurred at the University of Virginia (UVA) in late 2006, and in New Hampshire in June of 2006, one of which, FJ959107, was a imported case from Japan (Rota et al., 2009). The foreign JPC-1-related strains were phylogenetically distant from isolates of the 2006 large outbreak in Iowa, USA (JN012242), and related outbreaks in USA, Canada and Europe (Figure 7; Supplementary Figures S2, S3; Watson-Creed et al., 2006; Centers for Disease and Prevention, 2006a; Rota et al., 2009; Stapleton et al., 2019; Bodewes et al., 2020).

We also estimated the BSP-based effective population sizes for the JPC-1 viruses (Supplementary Figure S4; Supplementary Table S3). The results of the BSP analysis showed that the JPC-1 viruses have rapidly increased their population in the end of 1990s when right after the first JPC-1 isolate was detected, then have been gradually increasing until 2017.

JPC-2 was the second clade that emerged in Japan in 2001. In this study, it was identified in only two cases from Aichi and Mie prefectures (MuVi/Aichi#10879.JPN/6.01[G] and MuVi/Mie#W12.JPN/20.01[G]), the central region of Japan. However, as closely related strains were isolated in Hokkaido, the northernmost prefecture in 2000 or 2001 (AB105477, KF876710), and one of these, AB105477, shares a 100% identical sequence to MuVi/Aichi#10879.JPN/6.01[G], JPC-2 may have caused a nationwide epidemic in Japan within 2 years. The most closely related strain was isolated from a Burmese with recent travel to Myanmar, in Taiwan in 2007(MF281013; Supplementary Figure S3; Cheng and Liu, 2018). The tMCRA of JPC-2 was 1999.35 (95% HPD:2000.91–1996.41; Supplementary Figure S2; Supplementary Table S3).

**Figure 8:**
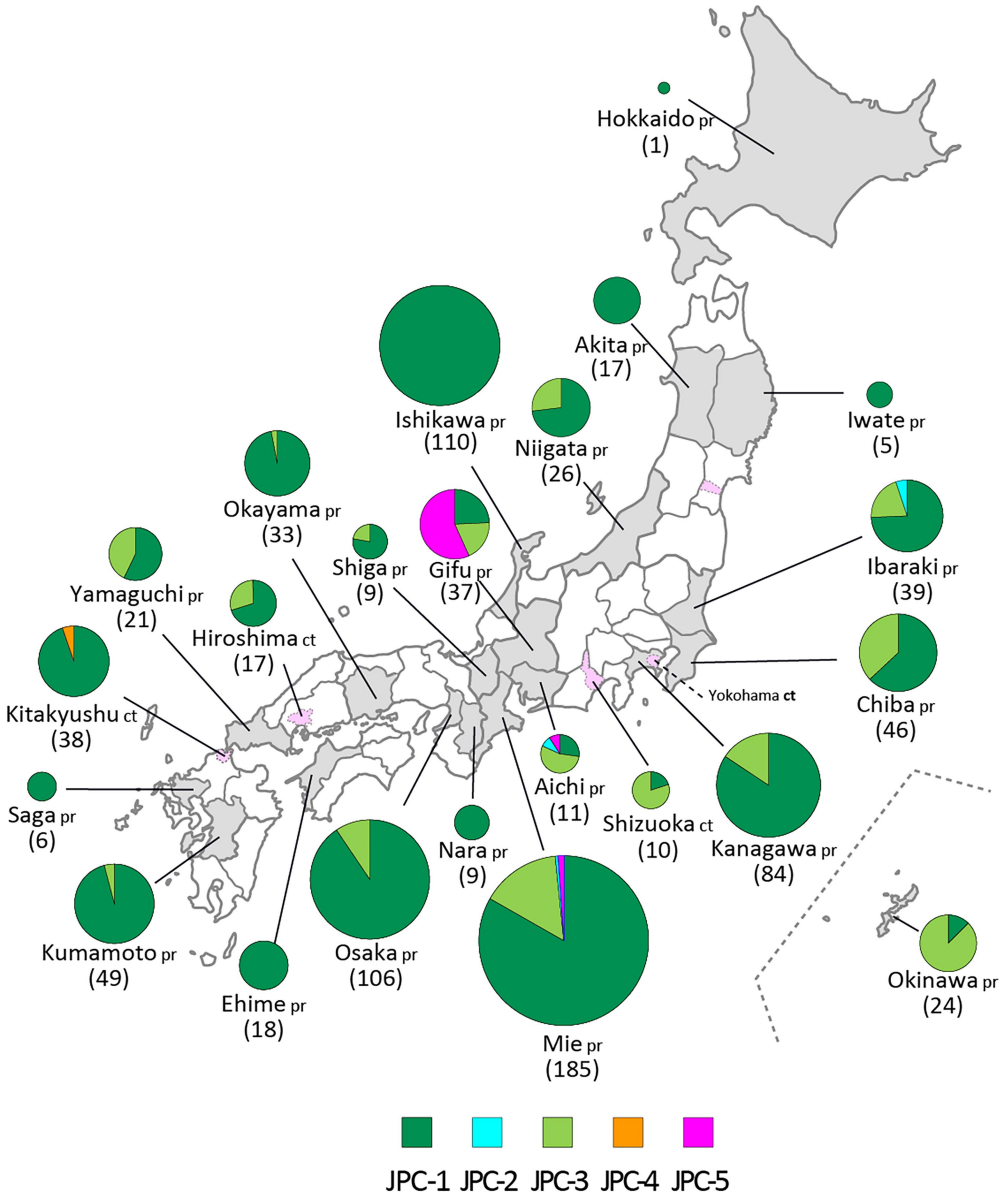
The geographic distributions of the Japanese clades of genotype G. The five clades of genotype G are color coded. The numbers in brackets refer to the total numbers of mumps viruses (MuVs) isolated from the prefectures (pr) or cities (ct). The administrative divisions where MuVs were isolated are color coded: grey-colored area for prefecture, pink-colored area surrounded by dashed line for city.

JPC-3, tentatively called “Ge,” was the third-emerged and second major clade of Japanese genotype G (144 isolates, 16.0% of genotype G), which has been co-circulating with JPC-1 for a decade. JPC-3 was first isolated from Niigata Prefecture at the end of 2009 (MuVi/Niigata#1604.JPN/49.09[G]). In some regions and periods, JPC-3 viruses predominantly circulated, such as Shizuoka City in 2010–2014, Aichi Prefecture in 2013–2016, and Okinawa main island in 2015–2016 (Kuba et al., 2017; Figure 8). In contrast, in some areas such as Osaka, Ibaraki, and Gifu prefectures, the two clades co-circulated in the same area during the same period. Closely related strains of the JPC-3 were also reported from Canada, United States, Taiwan, and China (MN910022, MN910184, JX455939, KF143753, MF281022, KF031051; Supplementary Figure S3). The tMCRA of JPC-3 was 2007.97(95% HPD: 2009.58–2006.07; Supplementary Figure S2; Supplementary Table S3).

The JPC-4 was singly isolated in Okinawa Prefecture in 2015 (LC167469; Kuba et al., 2017), and it was tentatively called “Ghk” because the isolate was phylogenetically most closely related to the Hong Kong isolate (KM234040) in 2014. In this study, two strains of JPC-4 were isolated successively in Kitakyushu City in 2016 (MuVi/Kitakyushu#062.JPN/31.16[G] and MuVi/Kitakyushu#082.JPN/34.16[G]), whose SH gene sequences shared 100% sequence similarity with that of the Okinawa isolate. Closely related foreign strains were isolated in the US and Canada along with China in 2014–2016 (KY013252, MN911765, MN910077, KM234040; Supplementary Figure S3). Of these, KY013252, the isolate from California, USA in 2016, was most homologous with JPC-4 strain (99.7% similarity). The remaining 3 strains, which shared 100% identical sequence, have 99.4% identity to JPC-4 strain. The tMCRA of JPC-4 could not be estimated because of the single WGS-dt sequence.

The JPC-5 was the latest clade of the Japanese genotype G (26 isolates, 2.9% of genotype G), which was first identified in 2016 in Mie, Gifu, and Aichi prefectures (MuVi/Mie#02.JPN/7.16[G], MuVs/Gifu#147.JPN/17.16[G], and LC424348). In 2017, in addition to Gifu Prefecture, the closely related isolates were identified in Ibaraki Prefecture (MuVs/Ibaraki.JPN/15.17[G] and MuVs/Ibaraki.JPN/25.17[G]) and Tokyo (LC325485; Figure 8). The Tokyo isolate shared a 100% identical SH gene sequence with Mie, Gifu, and Aichi isolates. Therefore, JPC-5 might have circulated over an extensive area of Japan in 2 years. Closely related strains were isolated in Canada and United States in 2015–2017 (MN926205, MF138973, KU508690, MH618522, MH618544; L’Huillier et al., 2018). Of these, a Canadian isolate (MN926205) shared the completely identical sequence with three Japanese isolates, MuVi/Mie 02.JPN/7.16[G], MuVs/Aichi.JPN/16.16[G], and LC325485 (Supplementary Figure S3). The tMCRA of JPC-5 was 2014.02(95% HPD: 2015.65–2011.83; Supplementary Figure S2; Supplementary Table S3).

### Diversities in the viral proteins of Japanese genotype G strains

In order to clarify the diversities of amino acid sequences within Japanese genotype G strains, the multiple alignments of all MuV proteins were constructed from the WGS sequences of 77 Japanese genotype G isolates (JPC-1, 64 strains; JPC-2, 2 strains; JPC-3, 8 strains; JPC-4, 1 strain; JPC-5, 2 strains; Supplementary Figure S5; Supplementary Table S4).

N protein is a major component of helical viral nucleocapsid and plays an important role in translation, transcription, and budding, where the characteristic “546DWD548” motif near the C-terminal of N protein plays an essential role for interaction with M protein and viral budding (Ray et al., 2016). N protein is also known as a target of the human CD8^+^ T cells (de Wit et al., 2019, 2020). The alignment of N protein shows that the DWD motif and human T cell epitope, N_115–122_, IPNARANL, are conserved among all Japanese genotype G strains along with three vaccine strains (Supplementary Figure S5; Supplementary Table S4).

The V protein blocks interferon (IFN) production and signaling pathway *via* its 69aa C-terminal cysteine-rich domain (CTD), which interacts with the host STAT (signal transducer and activator of transcription) molecules (Nishio et al., 2002), or the MDA5 (melanoma differentiation-associated gene 5; Andrejeva et al., 2004; Ramachandran and Horvath, 2010), the key molecules in IFN signal transduction pathway. This characteristic cysteine-rich motif, 170H-C-C-C-C-C-C217, was conserved in all strains along with three vaccine strains (Supplementary Figure S5).

The V/P gene gives rise to additional mRNAs by co-transcriptional addition of two non-templated G residues in the specific positions of mRNAs (referred to as RNA editing) resulted to generate the P proteins (Paterson and Lamb, 1990). Therefore, although V and P proteins share a common N-terminal 155-aa sequences, their C-terminal regions are different. The N-terminal common regions were known to harbor 5 experimentally characterized human T cell epitopes, V/P_17-25_, V/P _111–122_, V/P _142–150_, V/P _142–152_, V/P _144–152_ (Supplementary Figure S5; Supplementary Table S4). Of these, V/P_17-25_, which was one of the most broadly recognized by some human haplotypes, was conserved in all Japanese genotype G strains along with two Japanese vaccine strains, except for Jeryl Lynn vaccine strain (Supplementary Table S4). The remaining 4 epitopes were 100% conserved in JPC-2, -3, -4 and two Japanese vaccine strains, however, partially conserved in JPC-1 (84–97%) and JPC-5 (0 or 100%). The T cell epitope in V protein specific C-terminal region, V_194–202_, was conserved in the Japanese vaccine strains and JPCs, excepting JPC-1 (75%).

The P specific C-terminal region contains two T cell epitopes, P_235–243_, P_382–391_. P_235–243_ was 100% conserved in all Japanese genotype G strains along with all vaccine strains. P _382–391_ was conserved in the Japanese vaccine strains and JPCs, excepting JPC-1 (97%) (Supplementary Figure S5; Supplementary Table S4).

The M protein plays an essential role in the virion assembly and budding, by means of associating with the inner surface of the MuV envelope and the cytoplasmic tails of the envelope proteins, as well as the N proteins in the RNP complex. The sequence 24FPVI27 near the amino-terminal end of M protein plays an essential role for proper budding of MuV (Li et al., 2009). In the alignment of M protein, this motif was conserved in all 3 vaccine strains and JPC-3, JPC-4, JPC-5 strains (Supplementary Figure S5; Supplementary Table S4). However, interestingly, in three strains of JPC-1 and all 2 strains of JPC-2, this motif sequence was disrupted by V26I or I27V mutations. It is not clear how these mutations influence on budding process and pathogenicity of these mutant viruses. However, since physico- chemical properties of valine and isoleucine are similar, the influence of the mutations on viral replication could be restricted. M protein contains 10 T cell epitopes, M_108-116_, M_120-128_, M_161-169_, M_187-195_, M_303-312_, M_309-316_, M_337-345_, M_350-358_, M_364-372_, M_365-372_, all of which were conserved in the Japanese vaccine strains and isolates of all JPCs excepting for JPC-1. In JPC-1 strains, 8 of 10 epitopes, excepting M_108-116_, M_350-358_ were conserved. Meanwhile, in Jeryl Lynn stain, 5 of 10 epitopes, M_120-128_, M_161-169_, M_337-345_, M_350-358_, M_365-372_, were not conserved.

F protein, one of two envelope glycoproteins of MuV, is a target antigen of neutralization antibodies. The seven potential glycosylation sites and proposed three B-cell epitopes, 221I, 323 N, 373D (Santak et al., 2015) were conserved in all strains (Supplementary Figure S5; Supplementary Table S4). The polybasic cleavage site (99RxKR102, where x is any amino acid) recognized by cellular protease were conserved in JPC-1, -2, -5, and vaccine strains. Interestingly, in all strains of JPC-3 and -4, the basic amino acid, lysine (K) at position 101 were substituted to an analogous basic amino acid arginine (R) like as other paramyxoviruses, SV5 and RSV (Klenk and Garten, 1994). F protein contains 4 experimentally characterized T-cell epitopes, F_112-121_, F_152-161_, F_253-262_, F_445-453_, of which, all epitopes were conserved in two Japanese vaccine strains and JPCs excepting for JPC-1, even though its conserved ratios were high (84–100%; Supplementary Table S4). In Jeryl Lynn strain, a half of the epitopes were not identical.

HN protein, another envelope glycoprotein of MuV, plays a key role in the viral entry to the host cells *via* binding to the cellular receptor molecules, sialic acids (Kubota et al., 2016). After that, HN protein promotes a conformational change of F protein, resulted in a membrane fusion (Bose et al., 2014). The16 amino acid residues associated with receptor binding and 3 residues associated with fusion promotion by HN protein were conserved in all strains (Supplementary Figure S5). The eight potential glycosylation sites on HN ectodomain were all conserved. HN protein is the most important target antigen for neutralization antibodies, and at least three B-cell epitopes, HN_265–288_, HN_329–340_, HN_352–360_, were experimentally characterized (Orvell, 1984; Cusi et al., 2001; Kulkarni-Kale et al., 2007). The averages of the sequence homologies of the epitopes between Japanese vaccine strain Torii and JPC isolates were relatively higher (96–100%) than that between Jeryl Lynn strain and JPC isolates (88–92%). Notably, 4 experimentally identified T-cell epitopes in genotype G HN protein, HN_88-96_, HN_157-165_, HN_352–360_, HN_505-513_, were conserved in two Japanese vaccine strains and also highly conserved in JPCs (98–100%), while 2 of 3 epitopes were not conserved in Jeryl Lynn strain (Supplementary Figure S5; Supplementary Table S4).

L protein is the largest protein in the MuV viral components which has multifunctional enzymatic activities, in which 9 T-cell epitopes, L_249-256_, L_336-344_, L_441-449_, L_591-600_, L_740-748_, L_1299-1307_, L_1336-1343_, L_1806-1813_, L_1983-1992_, L_1986-1994_, were experimentally identified (de Wit et al., 2019; Kaaijk et al., 2021). All of them were conserved in two Japanese vaccine strains and almost all of JPC isolates (98–100%; Supplementary Figure S5; Supplementary Table S4). Meanwhile, 5 of 9 epitopes were not conserved in Jeryl Lynn strain.

In the study, truncated forms of mutant SH protein were identified in 7 strains, which coded three different length of SH proteins, 8-, 44-, and 50-aa (Supplementary Figure S5). These mutant SH proteins were generated through three types of nucleotide substitutions, C to A, C to T, or G to A, in different sites that generated new internal stop codons. These mutant viruses were all classified into JPC1, even though their phylogenetic distance and their isolation years (from 2006 to 2015) were not necessarily close (Figure 6; Supplementary Figure S5).

## Discussion

We collected 1,064 MuV sequences of mumps clinical samples and MuV isolates from 24 of 47 prefectures to investigate the nationwide molecular epidemiologic trend of MuV past three decades in Japan. We identified 6 genotypes, B, F, G, H, J, L, during the period (Figures 1, 3). Among them, genotype B, an indigenous genotype of Japan, was the second major genotype in the study (107 strains, 10.0%), which had been circulating until 2001 and finally replaced with genotype G (Figures 1A,B). Before the genotype G exclusively circulated, genotype B, J and L cocirculated during about 10 years since 1990s. Japanese genotype J and L strains are highly homologous, and they are phylogenetically divergent from the foreign related strains (Figures 5B,C). Unexpectedly, the Japanese genotype J strains are phylogenetically distant from the strains of neighboring Asian countries, like Taiwan, Hong-Kong and Thailand (Figure 5B). Regarding genotype L, we performed time-scaled Bayesian evolutionary analyses on the HN gene sequences of 11 strains. The results indicate that the mean tMRCA of Japanese clade was distant from that of European clade, and suggest that the Japanese genotype L strains could not be directly originated from the European genotype L strains (Supplementary Figures S1A,B, Supplementary Table S3). Therefore, the geographical origins of Japanese genotypes J and L remain unclear.

In 2000s, genotype G have driven away these genotypes and dominantly circulated over two decades in the nation. However, Japanese genotype G viruses were not homogeneous. At least 2 major clades consisting of 5 minor clades (JPC-1, -2, -3, -4, and -5) have emerged and some of them have cocirculated in some areas. After the first emergence of JPC-1 in 1997, JPC-2 and JPC-3 emerged in 2001 and 2009, respectively. The JPC-3 became a second major clade of the Japanese genotype G and cocirculated with JPC-1 over a decade in Japan. Lastly, the JPC-5 emerged in 2016 and caused epidemics mainly in a central region of Japan, until today.

The JPC-1strains, firstly emerged and most major clade of Japanese genotype G, were also isolated in foreign countries, United States and Canada (Supplementary Figure S3). However, since the isolation years of the foreign JPC-1 strains were all after the tMCRA of JPC-1, the JPC-1 viruses may not be originated from these areas. We also estimated the chronological change of effective population sizes of the JPC-1 viruses by using BSP analysis (Supplementary Figure S4), even though the result did not necessarily fit the real mumps epidemic curve in Japan (Figure 1C). One reason of that discrepancy could be due to biased sampling in time and area. However, these methods could be convenient approach for monitoring epidemic situations of mumps after introducing regular immunization program of MCVs in Japan by using WGS data.

Intriguingly, JPC-2 strains are phylogenetically close to the isolate from Taiwan in 2007 (MF281013: MuVs/Taipei.TWN/46.07[G]) which was obtained from a Burmese with recent travel to Myanmar (Cheng and Liu, 2018), whose homologies of SH gene sequences are between 99.1 to 99.4%. Therefore, JPC-2 viruses might have been imported from the area.

The first isolate of the JPC-3, MuVi/ Niigata #1604.JPN/49.09[G], shared the highly homologous (99.7%) SH sequences with the isolates from the large mumps outbreak of 2009–2010 in the US Territory of Guam (Mahamud et al., 2012; Nelson et al., 2013). In addition, the MuVs having the identical sequence with Guam isolates were also isolated from Hong Kong and Hawaii in 2009 and 2010, respectively, (KF031051, JX455939). It should be noted that the Hong Kong strain, KF031051, and the Niigata strain were isolated at almost the same time, on the 49th week in 2009. These results show that the JPC-3 viruses may originate from one of these areas, Hong Kong, Guam, or Hawaii. JPC-4 related foreign strains were isolated in Hong Kong, USA, and Canada in 2014–2016, of which the isolate from California, USA in 2016, was most homologous with JPC-4 strain. However, the first JPC-4 isolate was isolated in Ishigaki island of Okinawa prefecture in 2015, where a lot of tourists rushed from Hong Kong (Kuba et al., 2017), as is the case with Kitakyusyu city, where more than 1,800,000 tourists visited from Hong Kong in 2016. Therefore, JPC-4 viruses might have been imported from Hong Kong area, even though epidemiological links were unclear.

Regarding JPC-5, the most closely related strains were identified in Canada, United States and United Kingdom in 2010 and 2015 (KY006858, KU508690, MF522117). Therefore, the JPC-5 strains might be imported from these areas. On the other hand, two isolates from Ibaraki Prefecture (MuVs/Ibaraki.JPN/15.17[G], MuVs/Ibaraki.JPN/25.17[G]) were isolated from the unrelated returnees from Indonesia. In addition, a closely related Taiwan strain was isolated from a patient returned from Malaysia. Therefore, another possibility of the origin of the JPC-5 could be Southeast Asia area. However, it remains a possibility that the phylogenetic position of the Ibaraki strains among the Japanese genotype G can be inaccurate, because the phylogenetic results of the Ibaraki strains were obtained by only using SH-based analysis.

Meanwhile, the geographical origin of the JPC-1 viruses remains unclear, because the clade is phylogenetically distant from any other foreign genotype G sequences. In addition, it is not clear why genotype G has been circulating exclusively in Japan for over 15 years.

As described above, SH-based phylogenetic analysis is useful for predicting geographical origins of the circulating strains, because a large numbers of sequence data from around the globe are deposited in public databases. Meanwhile, SH gene-based analysis has critical limitations on the accuracy. It is fundamentally difficult to fit an appropriate substitution model to SH gene-base phylogenetic analysis, because multiple substitutions are likely to occur at the same points in a short and highly variable region like SH gene.

In contrast, WGS analysis is highly accurate, because the accuracy of the phylogenetic analysis improves as the length of the target sequence increases (Bodewes et al., 2020). The much higher bootstrap values of the WGS analysis than that of SH-base analysis provide strong evidence of the accuracy of the WGS analysis (Bryant et al., 2022).

In addition, WGS analysis can better identify genetic variations than an analysis by short single gene like SH gene, because the numerous variable sites can be identified by WGS (Houldcroft et al., 2017). Indeed, WGS-dt-based analysis can discriminate the isolates bearing completely identical SH sequence, which has been circulated over a period of 10 years in Mie Prefecture (Figure 7, Supplementary Figure S2). Therefore, WGS analysis can also be useful for tracing outbreak strains and distinguish them from the outbreak unrelated strains (Stapleton et al., 2019; Wohl et al., 2020; Bryant et al., 2022). In addition, by comparing amino acid sequences of MuV viral components of a circulating wild strain using WGS data, it would be possible to monitor any antigenic and pathogenic changes of circulating viruses.

In the comparison of amino acid sequences of all viral proteins among JPC viruses and vaccine strains by multiple alignments, it is noteworthy that the experimentally identified T-cell epitopes of genotype G virus were all conserved in Japanese vaccine strains (Supplementary Figure S5; Supplementary Table S4). In contrast, in Jeryl Lynn strain, only 20 (51%) epitopes were conserved. Although contributions of cellular immunity in mumps vaccine efficacy remain unclear, early studies have shown CD8^+^ T cells play a key role in viral clearance after many respiratory virus infections, and the presence of T cell response against MuV after vaccination and natural infection (de Wit et al., 2018, 2019; Schmidt and Varga, 2018). In addition, averages of the amino acid sequence homologies of the experimentally characterized B-cell epitopes between Japanese vaccine strain Torii and JPC isolates were relatively higher (96–100%) than that between Jeryl Lynn strain and JPC isolates (88–92%). Therefore, our data suggest that the probability of reduction of vaccine efficacy in Japanese vaccine strains due to the antigenic mismatch between current wild strains and vaccine strains might be lower than that in Jeryl Lynn strain.

Truncated forms of SH protein were detected in genotype G isolates in our study from Okayama, Mie, Kumamoto, and Kanagawa Prefecture from 2006 to 2015. Atypical SH gene mutants were previously detected in genotype G strains in United Kingdom (Cui et al., 2009) and a genotype C strain in India (KC429766; Jin et al., 2015). These data suggested that an SH gene is not essential for MuV infectivity, even though there are reports that the SH protein inhibits the TNF-α mediated apoptosis pathway (Wilson et al., 2006; Fuentes et al., 2007). Meanwhile, it may be noteworthy that the strains bearing truncated-form SH gene were all classified into genotype G, in particular into JPC-1.

We employed the RNA sequencing approach for WGS analysis as it is much more time- and labor-saving than the Sanger method. However, in this study, WGS analyses were applied only on the culture fluids of MuV isolates, as the RNA sequencing method requires a high copy number of viral genomes. We need to establish a method directly applicable to clinical specimens with a low copy number of MuV viral genomes.

The limitations of this study are that the background information of the clinical samples in this study, such as their epidemiological backgrounds, were limited. This made it difficult to precisely trace the epidemiological links of mumps transmissions and the geographic origins of MuV isolates in this study. In addition, since our surveillance systems were not fully organized before early 2000s, we could not sufficiently chase the mumps epidemics during the study period. Subsequently, the time points and geographic points of sample collections were limited and biased.

Our study shows the long-term and nationwide molecular epidemiology of MuV in Japan. These results provide baseline information about Japanese epidemic situation of MuV domestic genotypes, which is essential for evaluating the mumps elimination progress in Japan after the forthcoming introduction of the mumps vaccine into Japan’s regular immunization program. This study also shows that the NGS analysis is a useful tool for precise molecular epidemiology studies.

## Members of the surveillance team of mumps virus in Japan

National Institute of Infectious Diseases: Toru Kubota. Sendai National Hospital: Hidekazu Nishimura. Chiba Pref. Inst. of P.H.: Atsushi Ogura. Yamaguchi Pref. Inst. of P.H.E.: Shoichi Toda, Kaori Kuniyoshi. Niigata Pref. Inst. of P.H.E.S.: Tsutomu Tamura. Chiba City Inst. of H.E.: Toshimitsu Tanaka. Shizuoka City Inst. of Environ. Sci. and P.H.: Nona Shibahara, Michiko Asanuma, Takaharu Maehata. Aichi Pref. Inst. of P.H.: Yoshihiro Yasui. Shiga Pref. Inst. of P.H.: Kaori Yamada, Yuya Kosuge, Ryoko Yonetani, Yusuke Sugiki, Kayo Aoki. Osaka Inst. of P.H.: Yasutaka Yamanaka. Kitakyushu City Inst. of E.S.: Tatsumi Murata, Shinichi Seto, Misato Tachibana, Asuka Kikuchi. Kumamoto Pref. Inst. of P.H. and E.S.: Kenta Yoshioka. Okinawa Pref. Inst. of H.E.: Minoru Nidaira. Sendai City Inst. of P.H.: Ayumu Nakata, Yu Watanabe, Junko Mori.

## Data availability statement

The datasets of the MuVs isolated in the study are deposited in the DNA Data Bank of Japan (DDBJ). The list of the representative MuVs in this study and their accession numbers are provided in Supplementary Table S1, S2.

## Ethics statement

The protocols of this study and the publication of the data in academic journals was agreed by the Ethics Committee of the National Institute of Infectious Diseases (approval number 475, 738, 973).

## Author contributions

MK and MT designed the study. MK, TY, EN, HK, KN, TSan, KG, TK, TM, YK, TMu, WS, TH, NK, TSak, SY, AN, KW, CH, HH, YF, MY, HY, MS, HS, CS, MI, MF, HM, MI, AS, KM, HK, FK, and SS isolated MuVs and analyzed them. MK and TS reanalyzed the data and wrote the manuscript. All authors contributed to the article and approved the submitted version.

## Funding

This study was supported by a grant from the Ministry of Health, Labor, and Welfare of Japan and by a Grant-in-Aid from the Japan Agency for Medical Research and Development (AMED) under Grant Numbers JP18fk0108066, JP18fk0108014, JP19fk0108087, JP21fk0108612, and JP22fk0108646.

## Conflict of interest

The authors declare that the research was conducted in the absence of any commercial or financial relationships that could be construed as a potential conflict of interest.

## Publisher’s note

All claims expressed in this article are solely those of the authors and do not necessarily represent those of their affiliated organizations, or those of the publisher, the editors and the reviewers. Any product that may be evaluated in this article, or claim that may be made by its manufacturer, is not guaranteed or endorsed by the publisher.

## Footnotes

^1^ http://beast.bio.ed.ac.uk/Tracer

^2^ https://www.niid.go.jp/niid/images/iasr/rapid/mun/160413/munnen_190117.gif
