# Supplementary figures and images for: Nationwide and long-term molecular epidemiologic studies of mumps viruses that circulated in Japan between 1986 and 2017
Source: Front Microbiol. 2022 Oct 28;13:728831. doi: 10.3389/fmicb.2022.728831 (PMC9650061; doi:10.3389/fmicb.2022.728831)

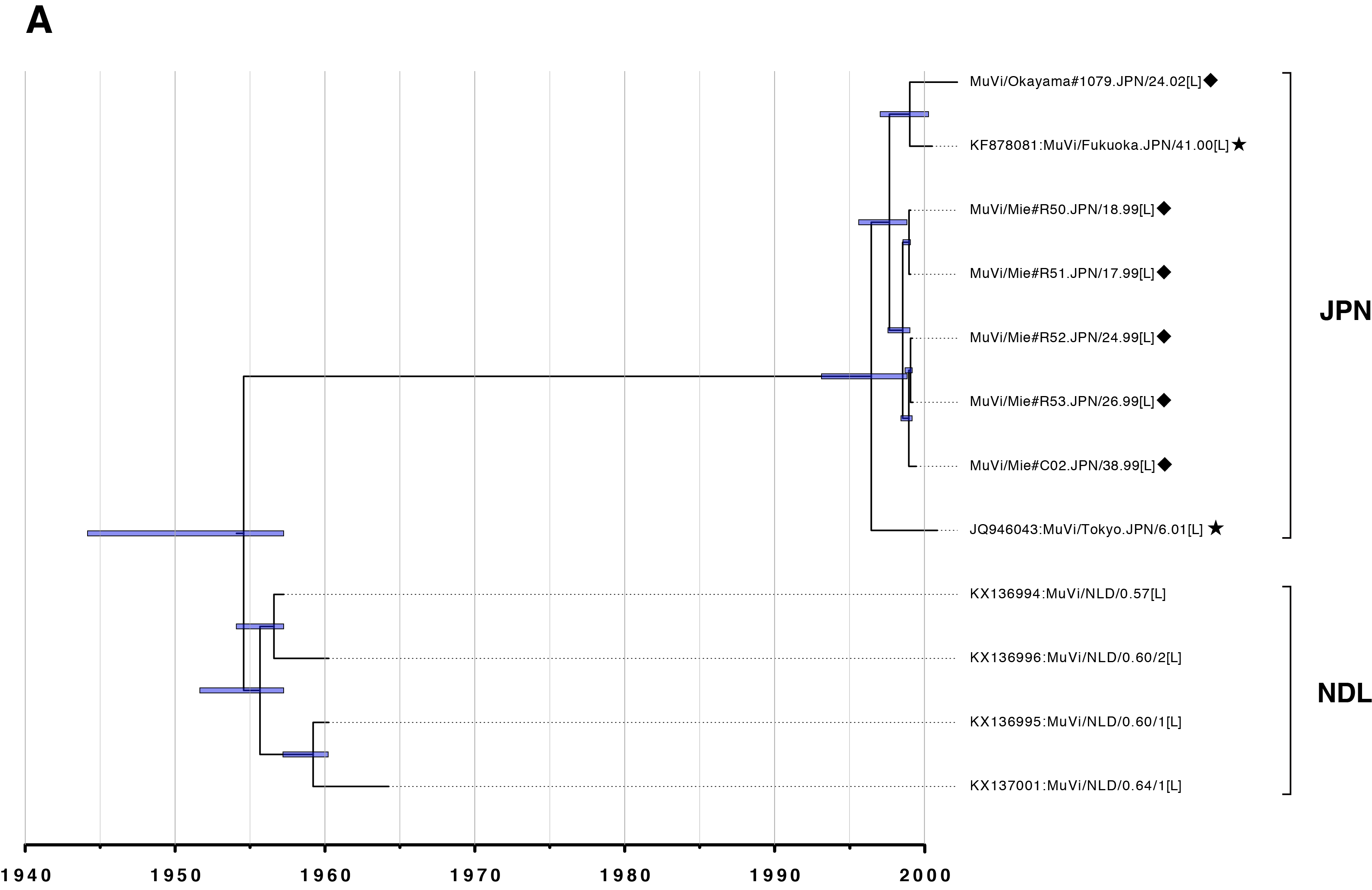

Supplement: Supplementary file 1 [file Image_1.jpg]

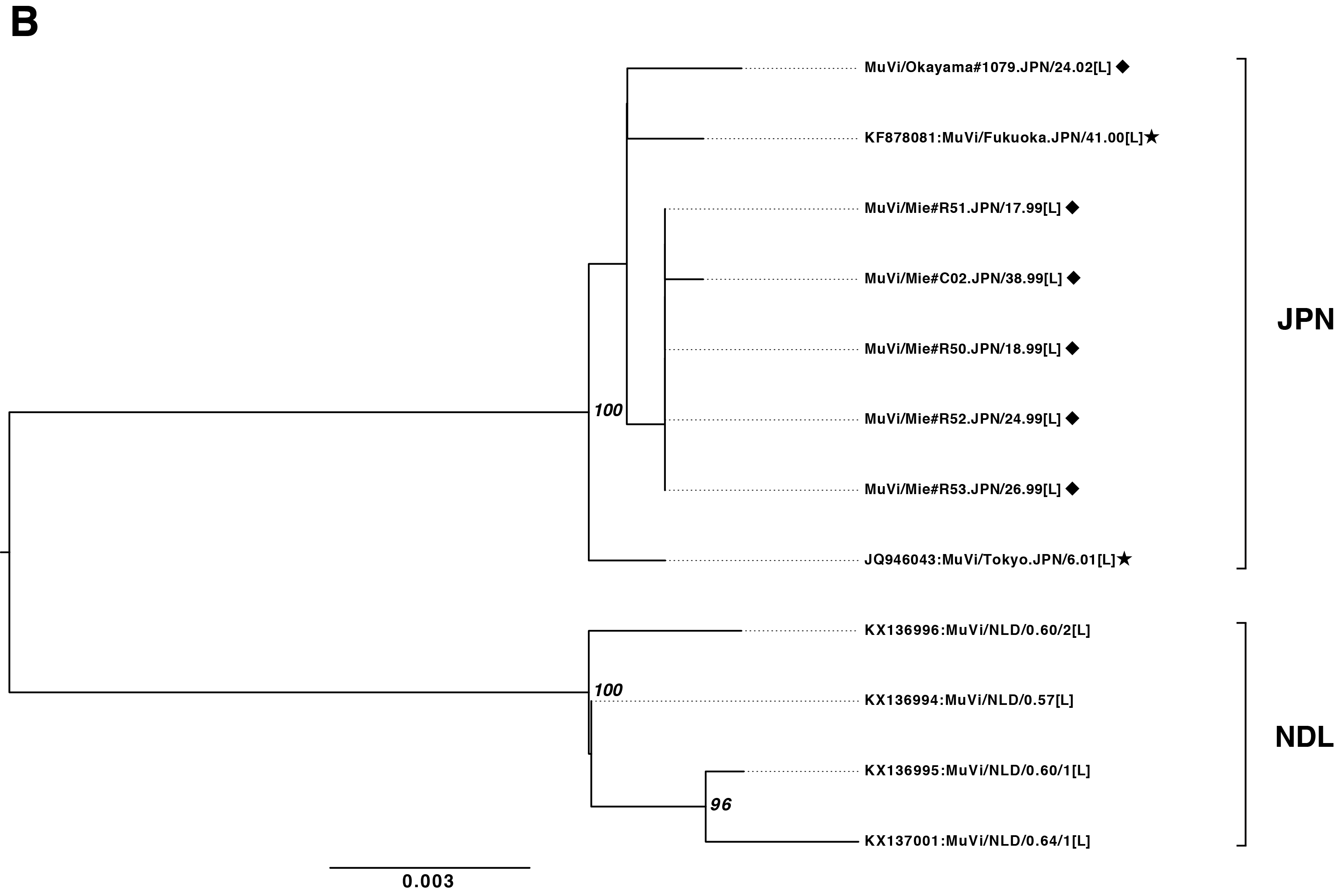

Supplement: Supplementary file 2 [file Image_2.jpg]

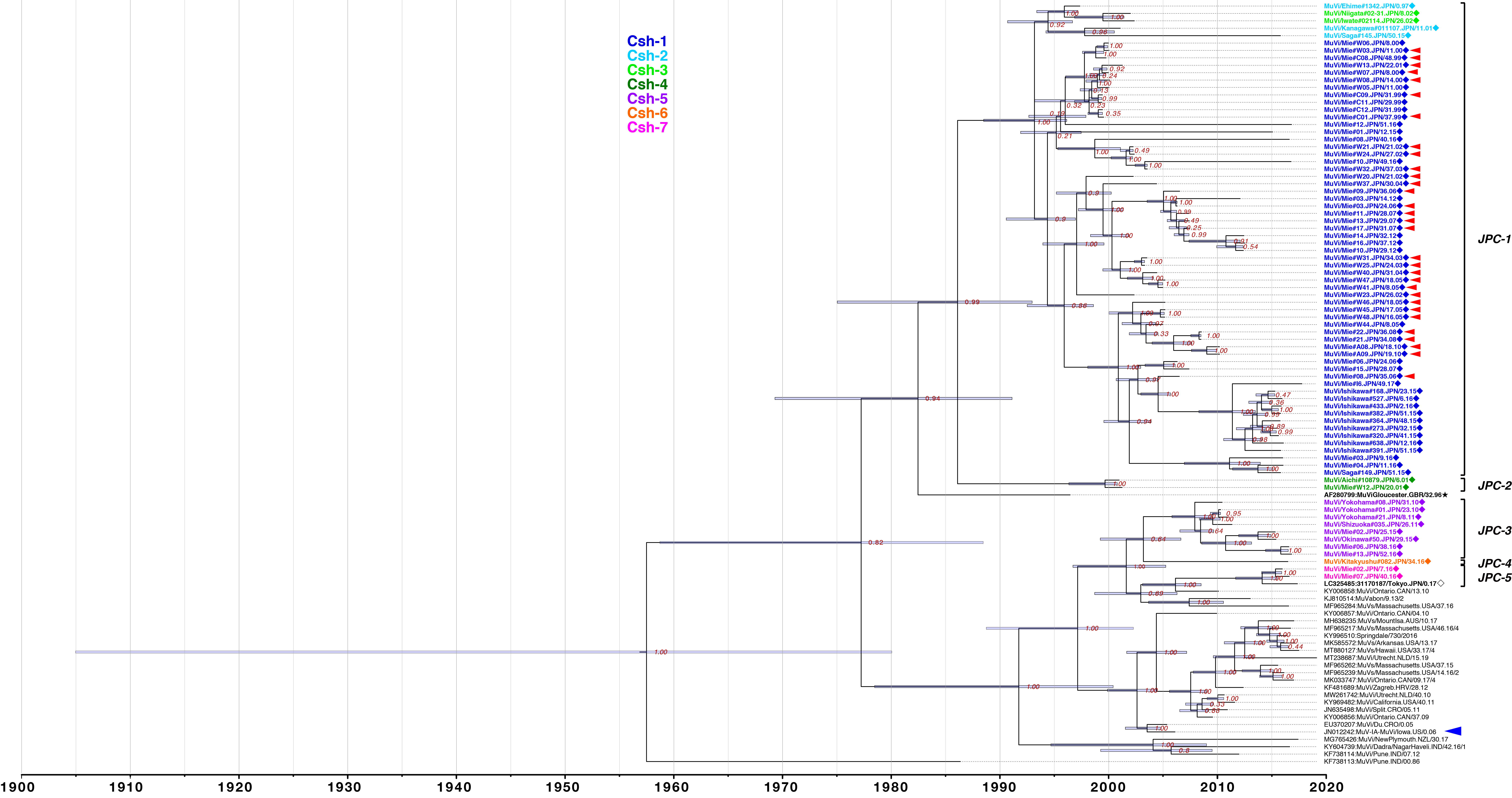

Supplement: Supplementary file 3 [file Image_3.jpg]

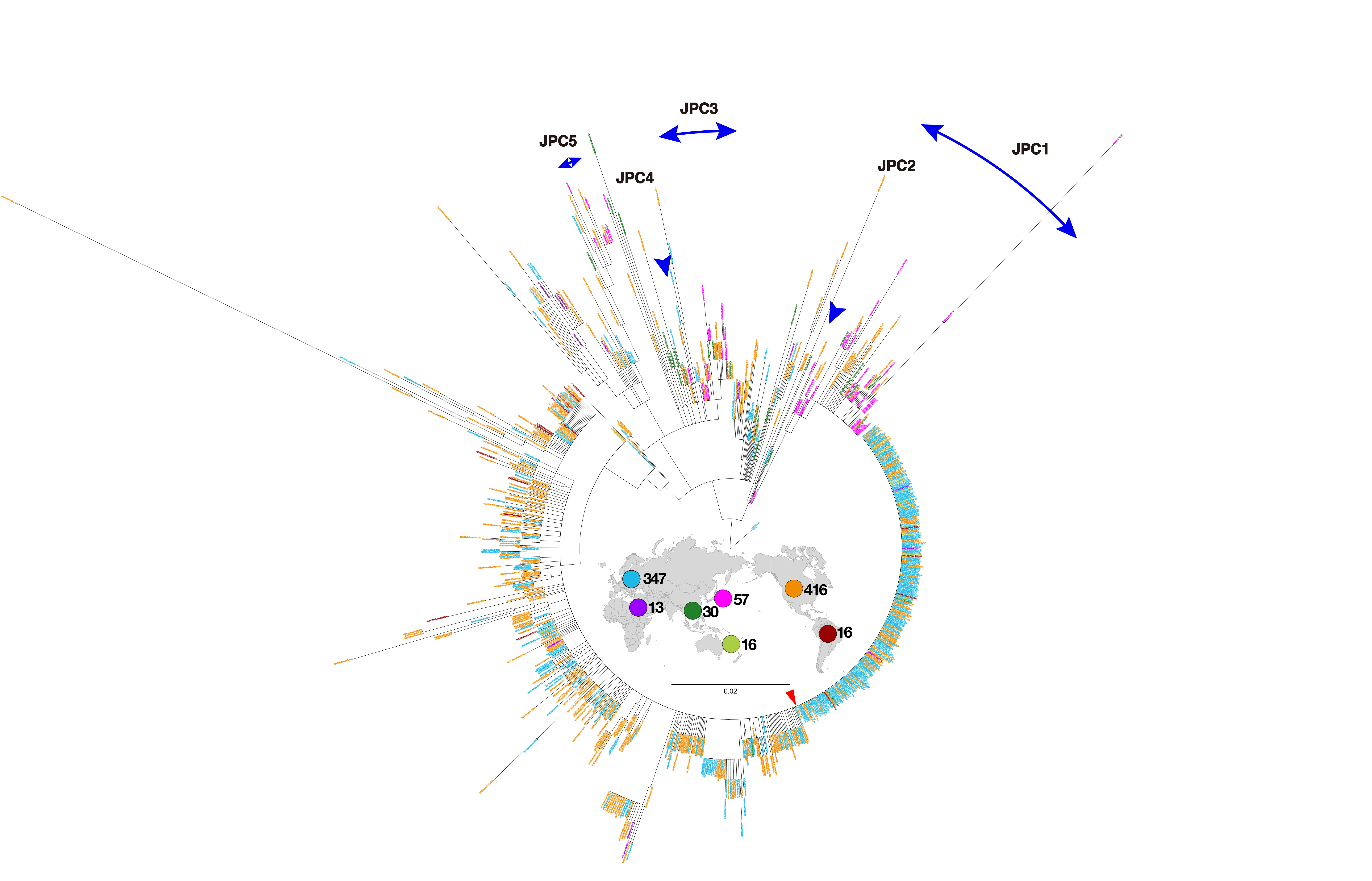

Supplement: Supplementary file 4 [file Image_4.JPEG]

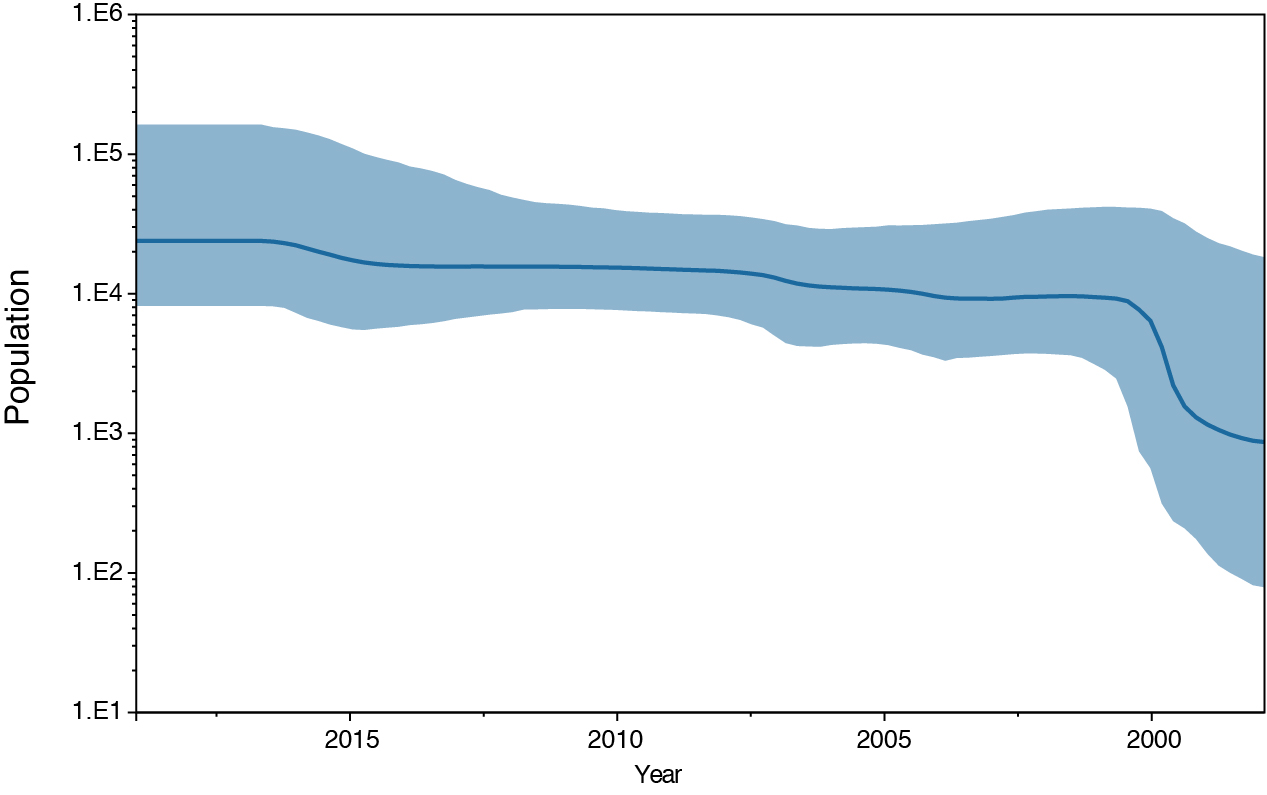

Supplement: Supplementary file 5 [file Image_5.JPEG]
