# Supplementary material for: Nationwide and long-term molecular epidemiologic studies of mumps viruses that circulated in Japan between 1986 and 2017
Source: Front Microbiol. 2022 Oct 28;13:728831. doi: 10.3389/fmicb.2022.728831 (PMC9650061; doi:10.3389/fmicb.2022.728831)
Supplement: Supplementary file 8 [file Table_3.DOCX]

**Supplementary Table S3**

**Mean times of the most recent common ancestor (tMRCAs) of genotype L and G isolates**

| Genotype | Clade | Date | | | Year | | | Posterior  probability |
| --- | --- | --- | --- | --- | --- | --- | --- | --- |
|  |  | Mean tMRCA | 95% HPD L | 95% HPD H | Mean tMRCA | 95% HPD L | 95% HPD H |  |
| L | JPN | 1996/9/12 | 1999/2/6 | 1993/5/18 | 1996.70 | 1999.10 | 1993.38 | 1.00 |
|  | NLD | 1955/12/2 | 1957/7/1 | 1951/11/28 | 1955.92 | 1957.50 | 1951.91 | 0.34 |
|  | root | 1954/10/30 | 1957/6/28 | 1944/5/29 | 1954.83 | 1957.49 | 1944.41 | 1.00 |
| G | JPC-1 | 1992/11/1 | 1996/4/4 | 1988/8/11 | 1992.84 | 1996.26 | 1988.61 | 1.00 |
|  | JPC-2 | 1999/5/8 | 2000/11/28 | 1996/5/28 | 1999.35 | 2000.91 | 1996.41 | 1.00 |
|  | JPC-3 | 2007/12/21 | 2009/8/1 | 2006/1/24 | 2007.97 | 2009.58 | 2006.07 | 1.00 |
|  | JPC-4 | - | - | - | - | - | - | - |
|  | JPC-5 | 2014/1/6 | 2015/8/26 | 2011/10/29 | 2014.02 | 2015.65 | 2011.83 | 1.00 |
