# Supplementary material for: Nationwide and long-term molecular epidemiologic studies of mumps viruses that circulated in Japan between 1986 and 2017
Source: Front Microbiol. 2022 Oct 28;13:728831. doi: 10.3389/fmicb.2022.728831 (PMC9650061; doi:10.3389/fmicb.2022.728831)
Supplement: Supplementary file 10 [file Table_5.docx]

**sFig-1.** Bayesian maximum clade credibility (BMCC) tree with mean node heights (A) and Maximum likelihood tree (B) based on the HN gene sequences of genotype L strains. The World Health Organization (WHO) reference strains are indicated by stars (★). The strains collected in this study are indicated by a filled rhombus (◆). **A:** Blue bars indicate 95% high probability distribution for the node height estimate. **B:** Bootstrap values >75% are indicated on the trees.

**sFig-2.** BMCC tree of the WGS-dt dataset of genotype G MuVs. The BMCC tree was inferred using the Bayesian analysis of the genotype G MuVs with the best fit model ( relaxed clock gamma distribution model). The 95% highest probability density (95%HPD) for each node age are shown as blue bars. The World Health Organization (WHO) reference strains are indicated by stars (★). The strains collected in this study are indicated by a filled rhombus (◆). The domestic related strains are indicated by an empty rhombus (◇). Red arrow-heads denote the strains harboring an absolutely identical SH gene sequence. Blue arrow-head denotes the isolate of the large outbreak in Iowa, USA in 2006.

**sFig-3.** Maximum likelihood tree of genotype G MuV was constructed based on the SH gene dataset of 30 Japanese strains and 867 representative strains retrieved from NCBI database. Blue double-headed arrows and arrow-heads indicate the strains of JPCs. Red arrow-head indicates the strain of the 2006 large outbreak in Iowa, USA. A world map is indicated with the locations and the numbers of the selected strains in the analysis. The isolated areas of MuVs are color-coded:( magenta for Japan, green for Asia excluding Japan, yellow-green for Oceania, blue for Europe, purple for Africa and Middle East, orange for North America, and brown for South America).

**sFig-4.** Bayesian Skyline plot (BSP) of the WGS-dt dataset of Japanese genotype G MuVs. Bayesian coalescent inference of genetic diversity and population dynamics using the BSP available in BEAST 1.6.1. for JPC1 MuV strains. The x-axis represents years and the y-axis represents the relative genetic diversity product of the effective population size. Blue line indicates the mean estimate and the blue shadow indicates the 95%HPD.

**sFig-5.** Multi-alignments of deduced amino acid (aa) sequences of 8 mumps virus (MuV) proteins of 77 (JPC-1, 64 strains; JPC-2, 2 strains; JPC-3, 8 strains; JPC-4, 1 strain; JPC-5, 2 strains) Japanese genotype G MuV isolates along with two current Japanese vaccine strains (Torii and Hoshino). The identical sequences of a viral protein of each JPC were represented by the sequence of the oldest isolate of each JPC. Dots represent aa residues identical to those of the Torii vaccine strain. Magenta-colored rectangles exhibit experimentally characterized CD8^+^ T cell epitope candidate regions of genotype G MuV that were reported in an earlier study (de Wit et al., 2020). Potential glycosylation sites are marked by orange rectangles.

N protein: Red-colored aa residues (546DWD548) exhibit the motif essential for virus-like particle production (Ray et al., 2016).

V protein: Rectangle exhibits the region of cysteine- rich C-terminal domain (CTD), which involves in the degradation of signal transducer and activator of transcription (STAT) proteins (Ramachandran and Horvath, 2010). Red-colored residues exhibit the highly conserved amino acids in CTDs among paramyxoviruses.

M protein: Red-colored 4 residues (24FPVI27) exhibit the motif essential for efficient MuV virion production and budding.

F protein: Red-colored aa residues (99R-KR102) indicate a polybasic cleavage site recognized by cellular protease. Blue-colored rectangles indicate the B-cell epitopes suggested in earlier study (Santak et al., 2015).

HN protein: Black open square indicates a transmembrane domain. Blue-colored rectangles are indicated the epitopes of neutralizing antibodies as suggested in earlier studies (Orvell, 1984; Kovamees et al., 1990; Kulkarni-Kale et al., 2007). Green-colored residues indicates the residues associated with triggering F protein activation (Bose et al., 2014). Red-colored residues indicates the residues associated with receptor binding (Kubota et al., 2016). Yellow rectangle indicates the residues associated with a neuraminidase activity (Mirza et al., 1994).

SH protein: Hyphen represents a deleted aa residue.

**References**

Bose, S., Song, A.S., Jardetzky, T.S., and Lamb, R.A. (2014). Fusion activation through attachment protein stalk domains indicates a conserved core mechanism of paramyxovirus entry into cells. *J Virol* 88(8)**,** 3925-3941. doi: 10.1128/JVI.03741-13.

de Wit, J., Emmelot, M.E., Meiring, H., van Gaans-van den Brink, J.A.M., van Els, C., and Kaaijk, P. (2020). Identification of Naturally Processed Mumps Virus Epitopes by Mass Spectrometry: Confirmation of Multiple CD8+ T-Cell Responses in Mumps Patients. *J Infect Dis* 221(3)**,** 474-482. doi: 10.1093/infdis/jiz480.

Kovamees, J., Rydbeck, R., Orvell, C., and Norrby, E. (1990). Hemagglutinin-neuraminidase (HN) amino acid alterations in neutralization escape mutants of Kilham mumps virus. *Virus Res* 17(2)**,** 119-129. doi: 0168-1702(90)90073-K [pii].

Kubota, M., Takeuchi, K., Watanabe, S., Ohno, S., Matsuoka, R., Kohda, D., et al. (2016). Trisaccharide containing alpha2,3-linked sialic acid is a receptor for mumps virus. *Proc Natl Acad Sci U S A* 113(41)**,** 11579-11584. doi: 10.1073/pnas.1608383113.

Kulkarni-Kale, U., Ojha, J., Manjari, G.S., Deobagkar, D.D., Mallya, A.D., Dhere, R.M., et al. (2007). Mapping antigenic diversity and strain specificity of mumps virus: a bioinformatics approach. *Virology* 359(2)**,** 436-446. doi: 10.1016/j.virol.2006.09.040.

Mirza, A.M., Deng, R., and Iorio, R.M. (1994). Site-directed mutagenesis of a conserved hexapeptide in the paramyxovirus hemagglutinin-neuraminidase glycoprotein: effects on antigenic structure and function. *J Virol* 68(8)**,** 5093-5099. doi: 10.1128/JVI.68.8.5093-5099.1994.

Orvell, C. (1984). The reactions of monoclonal antibodies with structural proteins of mumps virus. *J Immunol* 132(5)**,** 2622-2629.

Ramachandran, A., and Horvath, C.M. (2010). Dissociation of paramyxovirus interferon evasion activities: universal and virus-specific requirements for conserved V protein amino acids in MDA5 interference. *J Virol* 84(21)**,** 11152-11163. doi: 10.1128/JVI.01375-10.

Ray, G., Schmitt, P.T., and Schmitt, A.P. (2016). C-Terminal DxD-Containing Sequences within Paramyxovirus Nucleocapsid Proteins Determine Matrix Protein Compatibility and Can Direct Foreign Proteins into Budding Particles. *J Virol* 90(7)**,** 3650-3660. doi: 10.1128/JVI.02673-15.

Santak, M., Orvell, C., and Gulija, T.K. (2015). Identification of conformational neutralization sites on the fusion protein of mumps virus. *J Gen Virol* 96(Pt 5)**,** 982-990. doi: 10.1099/vir.0.000059.
