# Supplementary material for: Nationwide and long-term molecular epidemiologic studies of mumps viruses that circulated in Japan between 1986 and 2017
Source: Front Microbiol. 2022 Oct 28;13:728831. doi: 10.3389/fmicb.2022.728831 (PMC9650061; doi:10.3389/fmicb.2022.728831)
Supplement: Supplementary file 11 [file Table_6.DOCX]

Supplement Figure S5: Multiple alignments of MuV proteins of Japanese isolates.

**
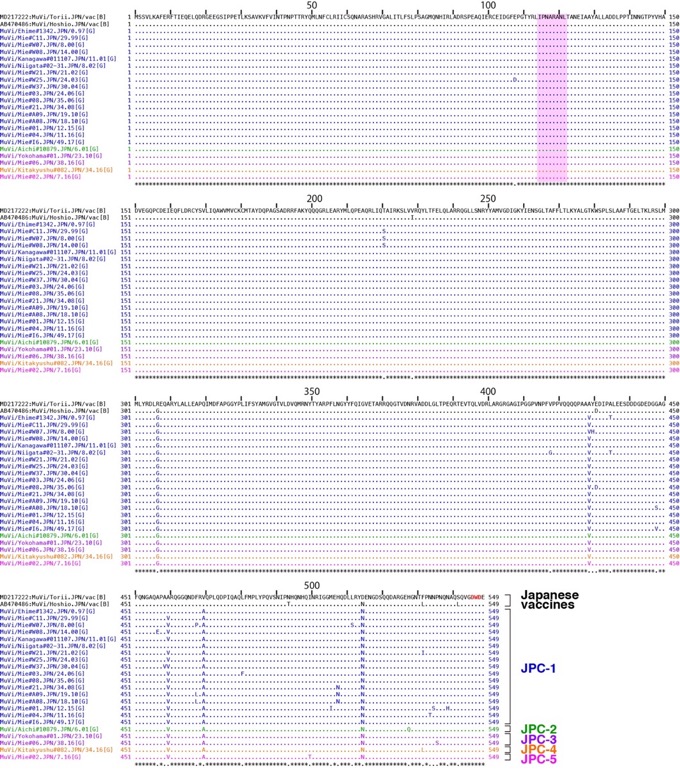
N protein**

**
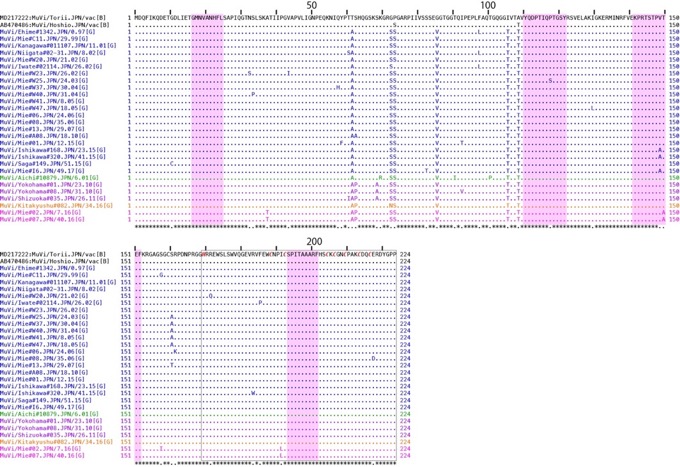
V protein**

**
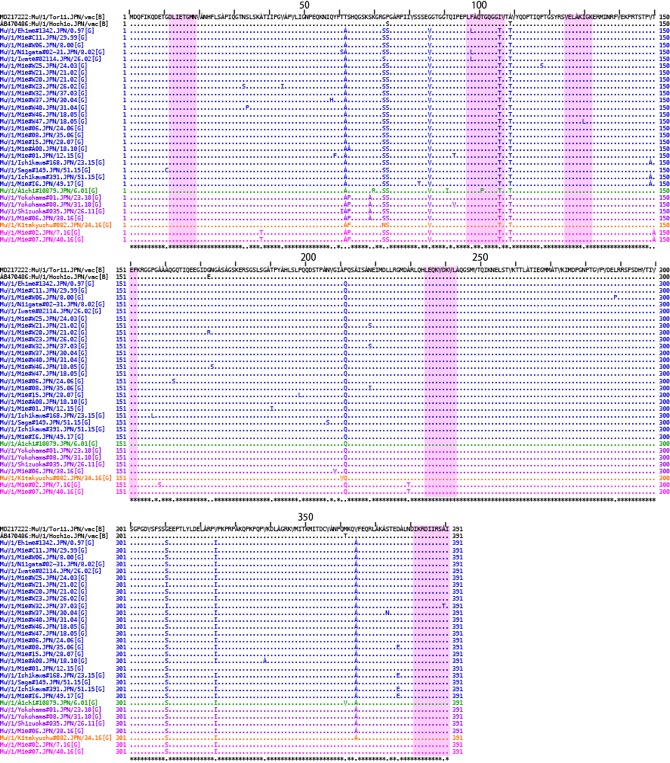
P protein**

**
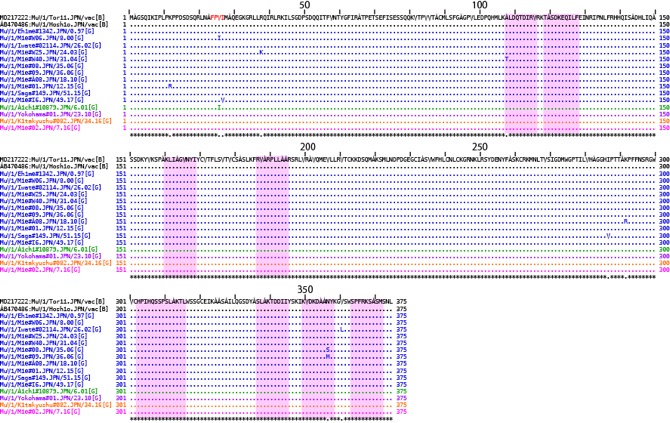
M protein**

**
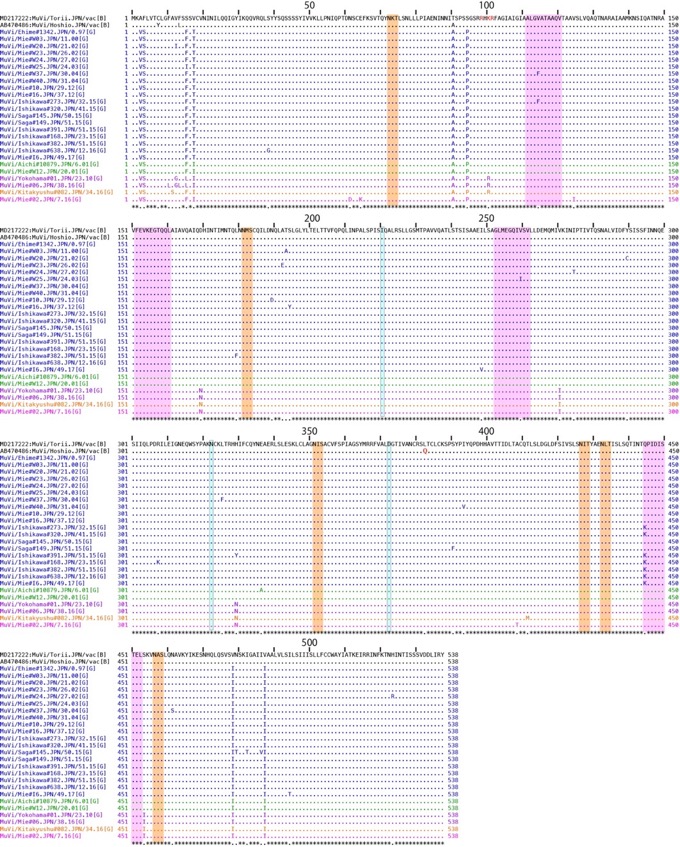
F protein**

**
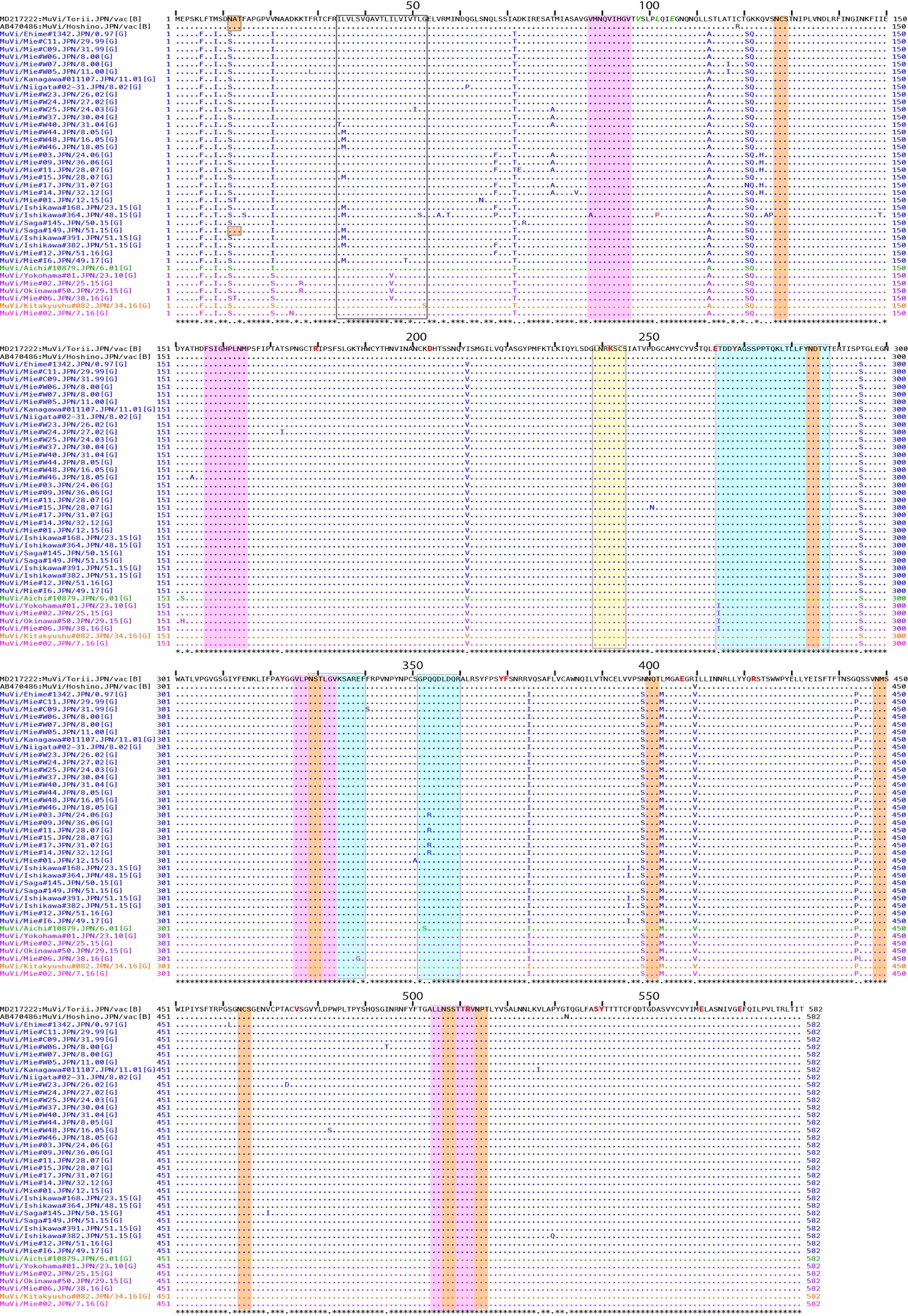
HN protein**

**L protein**

**
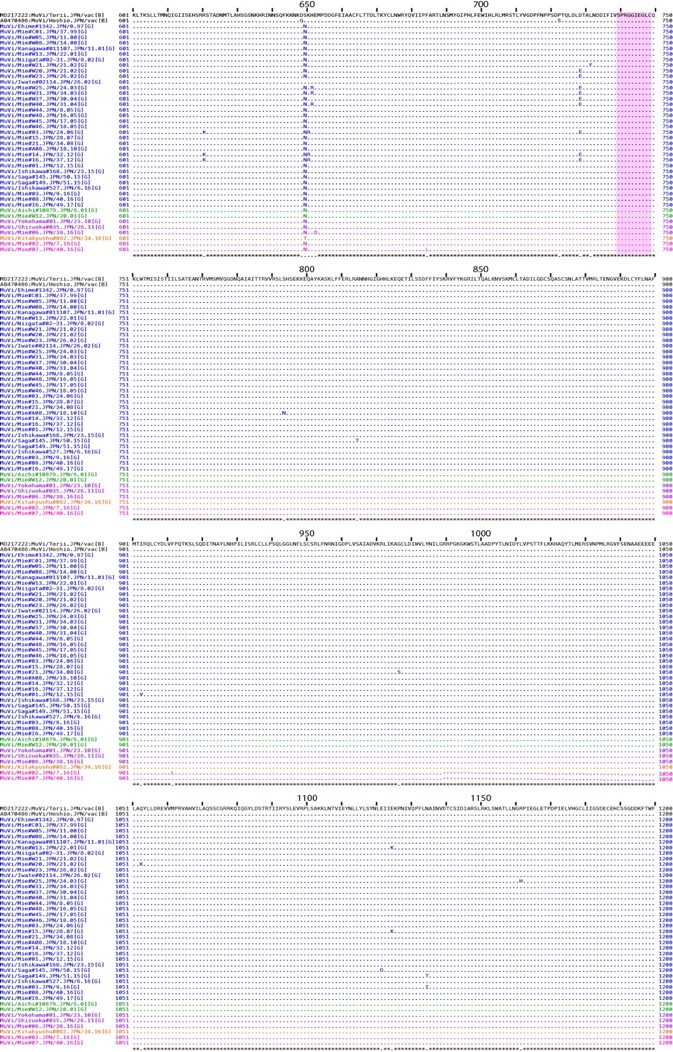
**

**
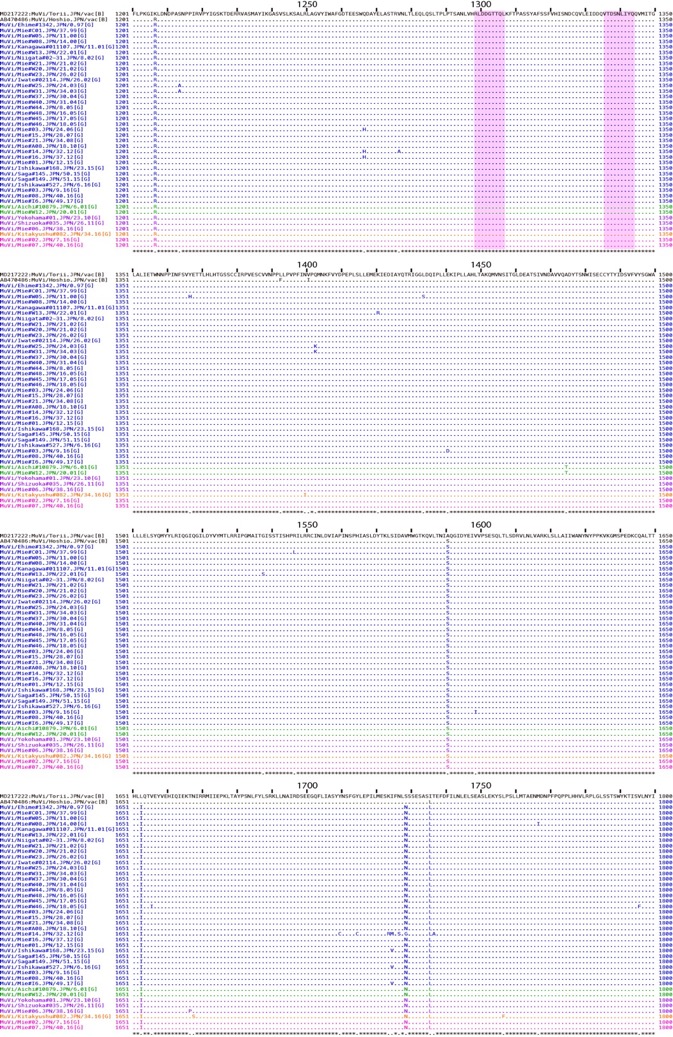
**

**
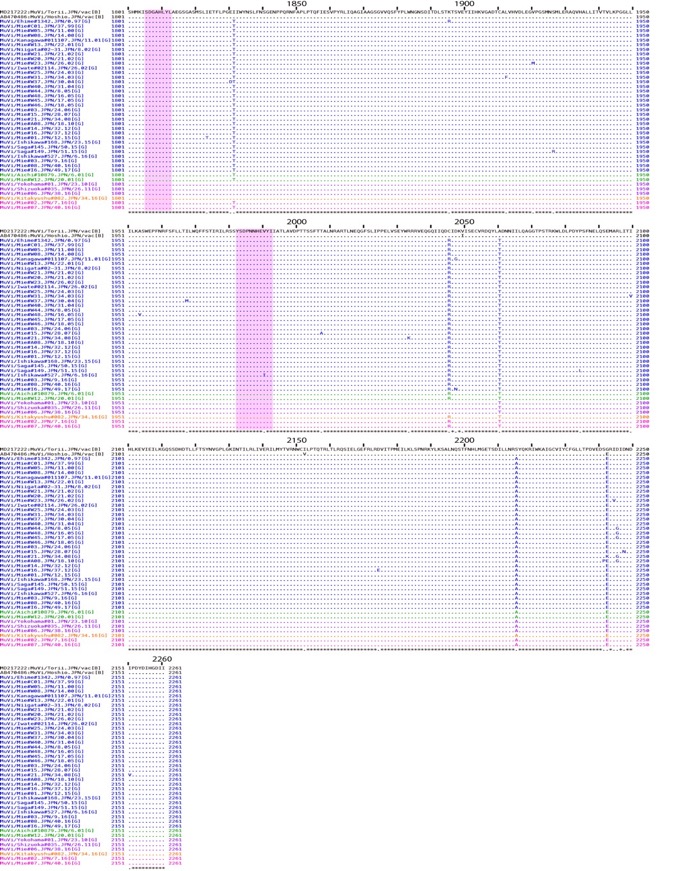
**

**SH protein**

**
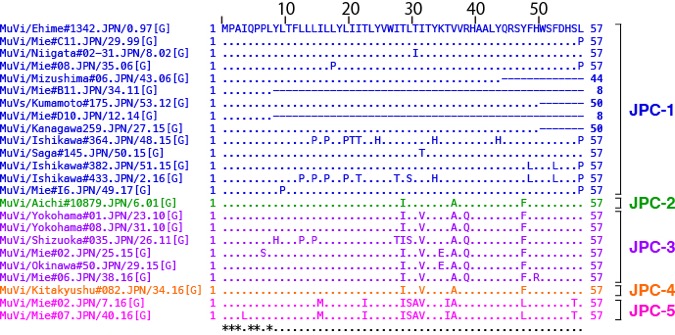
**
